# Behavioral decision-making and path optimization of stakeholders in straw collection, storage, and transportation within rural public health governance

**DOI:** 10.3389/fpubh.2026.1826051

**Published:** 2026-06-23

**Authors:** Hui Xu, Xiuli Duan, Yuling Xu, Tianlei Yan, Zhanwei Tian, Yun Teng

**Affiliations:** 1 School of Management, Trinity Western University, Langley Township, BC, Canada; 2 School of Information Engineering, Heilongjiang Forestry Vocation-Technical College, Mudanjiang, China; 3 College of Engineering, Northeast Agricultural University, Harbin, China

**Keywords:** behavioral strategy, evolutionary game theory, renewable resources, rural public health, straw collection, storage, and transportation (CS& T)

## Abstract

**Introduction:**

Efficient utilization of straw resources addresses both resource scarcity and environmental degradation while promoting green development. Despite China's abundant straw resources, uneven spatial distribution and practical challenges in collection, storage, and transportation (CS&T) limit large-scale utilization and pose rural public health risks due to improper disposal.

**Methods:**

This study integrates government, straw-specialized cooperatives, and farmers into a unified analytical framework and applies evolutionary game theory to examine strategic choices and dynamic interactions among stakeholders in the CS&T process. Key factors analyzed include government regulation costs, subsidy rewards, straw collection and transportation costs, and sales revenues.

**Results:**

The results indicate that subsidy rewards and sales revenues are the most significant drivers of stakeholder participation. Moderately increasing subsidies and rewards, while appropriately reducing fines, lowers participation costs for cooperatives and farmers, promoting active involvement and reducing open-air burning. Reducing government regulation costs, improving subsidy allocation accuracy, and moderately increasing fines enhance supervision efficiency. Stakeholder strategies are interconnected in a dynamic feedback system, forming a collaborative cycle of “field collection, centralized storage, standardized transportation, and resource recycling,” which improves CS&T efficiency.

**Discussion:**

Long-term policy effectiveness requires a scientifically designed incentive mechanism, with subsidies increased but capped at no more than twice the current standard, balancing motivation, fiscal sustainability, and policy feasibility. This study clarifies multi-stakeholder interaction mechanisms and offers theoretical and practical guidance for optimizing straw resource utilization and strengthening rural public health governance.

## 1 Introduction

Crop straw represents the most abundant and widely distributed renewable resource on Earth, globally recognized as the fourth-largest energy source after petroleum, coal, and natural gas (1, 2). As the primary carrier of photosynthetic products, straw sequesters more than half of the total photosynthetic accumulation generated during crop growth. It is highly enriched with essential macro-elements—including nitrogen, phosphorus, potassium, calcium, and magnesium—as well as vital trace elements such as iron and zinc (3). With an organic matter content exceeding 70%, straw serves as a high-quality renewable biomass resource, offering multifaceted value across ecological protection, resource recycling, and industrial value-added potential (4, 5). As a preeminent agricultural power, China consistently ranks among the world's leaders in total straw resources. Its distribution spans critical agricultural regions, including the Northeast Songliao Plain, the Huang-Huai-Hai Plain, and the middle and lower reaches of the Yangtze River. China's annual straw output remains stable at approximately 864 million tons, accounting for nearly 20% of global production. This vast resource is more than a mere byproduct of cultivation; it is a fundamental pillar for the green development of agriculture (6). Consequently, the efficient utilization and scientific disposal of straw are imperative—not only for enhancing resource recycling efficiency and extending agricultural value chains but also for fortifying ecological security barriers and ensuring long-term agricultural sustainability (7).

After years of policy guidance and practical exploration, China's comprehensive straw utilization has gradually formed a development pattern centered on “field return recycling as the primary approach, off-field industrial utilization as an effective supplement, and burning bans and control as the baseline.” The proportion of off-field straw utilization has steadily increased from less than 30% at the beginning of the 13th Five-Year Plan period to over 40% at present, demonstrating a favorable upward trend. At the national level, the government has continuously strengthened its policy support for off-field straw utilization through financial subsidies, project support, and technology promotion. However, due to the long-standing characteristics of China's agricultural production model, small-scale farming remains the dominant operational form (8), with approximately 90% of the country's arable land cultivated by smallholder farmers. This has resulted in a “scattered, widespread, and fragmented” distribution of straw resources (9). Furthermore, practical constraints such as insufficient mechanization in some remote areas and inadequate equipment for straw collection, storage, and transportation (CS&T) have reduced efficiency across the biomass supply chain, including storage, feeding, processing, and transportation. As a result, the CS&T process has become the central bottleneck restricting the large-scale and efficient utilization of straw (10, 11). Critically, an inadequate CS&T system not only leads to the waste of agricultural resources but also directly undermines the efficacy of rural public health governance. In areas lacking CS&T coverage, open-air burning and illicit piling remain rampant. The resulting emissions of particulate matter (PM2.5, PM10), carbon monoxide (CO), and volatile organic compounds (VOCs) constitute a major source of rural air pollution during the autumn and winter months (12, 13). In addition, randomly piled straw provides breeding grounds for mosquitoes, flies, and fungi, while leachate generated by rainfall may contaminate soil and water resources, posing significant threats to villagers' health, living environments, and rural public health safety (14, 15). Therefore, advancing the standardization, professionalization, and marketization of straw CS&T has become a critical pathway for improving rural public health governance, promoting rural ecological revitalization, and supporting the development of healthy rural communities.

Straw CS&T refers to the entire process of collecting and transporting straw from the field to storage facilities or directly to straw utilization enterprises as raw materials, while maintaining its utilization value. This process relies on economically efficient, timely, and convenient collection methods and equipment (16). Currently, the most widely adopted straw CS&T model in China is the cooperative-led third-party model. Under the guidance and promotion of government policies, social capital has been mobilized to support the establishment of farmer straw cooperatives. These cooperatives typically operate at the town or village level, setting up multiple straw collection and storage points to form a networked system. By signing collection agreements with farmers, they ensure the timely collection and centralized transportation of straw (10, 17). The involvement of straw cooperatives effectively substitutes for smallholder farmers in undertaking specialized tasks such as straw bundling, collection, and transportation. Through formal or informal cooperation arrangements with farmers and straw utilization enterprises, cooperatives establish stable supply–demand linkages. More importantly, by strengthening trust among stakeholders and reducing risk perception, cooperatives not only promote the efficient development of straw off-field utilization networks (18), but also mitigate practices such as open-air burning and random piling through centralized and standardized straw management. In doing so, they help address public health risks associated with straw-related pollution at the source and provide an important practical foundation for rural public health governance.

For both cooperatives and farmers, cost control is a core objective in profit maximization (19). However, the high costs and low returns associated with the straw CS&T process often result in insufficient motivation among participants, which in turn affects the coverage of standardized straw management and off-field utilization, indirectly weakening the effectiveness of rural public health governance. To ensure reasonable returns for stakeholders involved in straw off-field utilization, stimulate their participation in straw collection and transportation, and strengthen rural public health protection, the government urgently needs to introduce targeted incentive policies. Measures such as precise subsidies should balance resource utilization efficiency with public health governance objectives, while ensuring the stability of straw raw material supply and the standardization of straw management. In fact, at the national level, a policy framework for straw burning control and off-field utilization has already been established. The *Air Pollution Prevention and Control Law of the People's Republic of China* clearly defines areas where open burning of straw is prohibited and sets fines and other penalties for violations, aiming to curb the source of straw pollution at the legal level. The *State Council's General Office's Opinions on Accelerating the Comprehensive Utilization of Crop Straw* explicitly prioritizes the ban on straw burning and comprehensive utilization of remaining straw, aiming to establish a well-structured industrialization model for straw utilization. As early as 2015, the National Development and Reform Commission and other four departments jointly issued the *Notice on Further Accelerating the Comprehensive Utilization of Crop Straw and the Ban on Burning Work* (NDRC Circular [2015] No. 2651), further clarifying support for the development of straw CS&T service organizations, the construction of standardized storage facilities, and the encouragement of enterprises and social organizations to form specialized CS&T institutions, as well as the involvement of social capital. This policy not only focuses on the efficient utilization of straw resources but also implicitly emphasizes the governance orientation of preventing rural public health risks through the standardized disposal of straw. Subsequent policies such as the *14th Five-Year Plan for Circular Economy Development* and the *Implementation Plan for Agricultural and Rural Carbon Emission Reduction and Carbon Sequestration* have continued to strengthen support for the ban on straw burning and off-field utilization, clarifying phased goals for the comprehensive utilization rate of straw, and providing a solid policy foundation for the construction of straw CS&T systems and public health risk prevention.

Driven by a series of policy initiatives, China has continuously strengthened the construction of the straw CS&T system. Measures such as fiscal subsidies, the establishment of key demonstration counties, the layout of specialized collection and storage sites, and the promotion of diversified off-field utilization models have accelerated the transition of straw utilization toward a more standardized and market-oriented pattern. In recent years, the comprehensive utilization rate of straw has continued to increase in major grain-producing regions, including Northeast China and North China. In some areas, an integrated resource utilization system covering the full chain of “collection–storage–transportation–utilization” has gradually emerged. These efforts have contributed to reducing open-field burning, promoting resource recycling, and improving rural living conditions. Despite these achievements, the overall straw CS&T system is still at an early stage of development. Problems such as difficulties in straw collection, storage, transportation, and subsequent utilization remain unresolved. Influenced by fragmented smallholder farming, high CS&T costs, unstable market returns, and weak coordination among multiple stakeholders, open-field burning and random straw dumping still occur in some regions. These practices not only reduce the efficiency of straw resource utilization, but also intensify rural air pollution and public health risks. Against this background, an important practical challenge is how to improve coordination among government guidance, the organizational support of straw-specialized cooperatives, and farmer participation. Effective collaboration among these actors is essential for ensuring the stable operation of the straw CS&T system and for promoting the joint improvement of resource governance and public health governance.

In light of this, existing studies still exhibit three major limitations. First, most previous research has focused on economic objectives such as the efficiency of straw resource utilization, cost control, and industrial chain coordination. Insufficient attention has been paid to the environmental and public health problems caused by open-field burning and random straw dumping during the straw CS&T process. As a result, an integrated analytical framework linking resource governance with public health governance has not yet been fully established. Second, although some studies have begun to apply evolutionary game theory to examine stakeholder interactions in agricultural waste management, most of them concentrate on straw incorporation, energy utilization, or industrial chain coordination. Research specifically targeting the straw CS&T process, which serves as a critical intermediate link, remains limited. In particular, there is still a lack of systematic explanation of the dynamic strategic interactions among governments, straw-specialized cooperatives, and farmers. Third, most existing evolutionary game studies rely on hypothetical parameters or experience-based assumptions in simulation analysis. Empirical support from real-world survey data is often insufficient. This limitation reduces the consistency between research findings and actual policy implementation scenarios.

The promotion of off-field straw utilization is not merely a technological or market issue in essence. Rather, it is a multi-stakeholder collaborative governance problem shaped by the interactions among government regulation, cooperative operations, and farmer behavior. Particularly under the background of fragmented smallholder farming, the strategic failure of any single stakeholder may disrupt the straw CS&T system, which can further trigger open-field burning, environmental pollution, and the spread of public health risks. Against this backdrop, a key question deserves further investigation: under conditions of bounded rationality, how do the strategic interactions among governments, straw-specialized cooperatives, and farmers affect the stable operation of the straw CS&T system? Moreover, how do different policy instruments influence the evolutionary paths of stakeholder behavior and ultimately affect the effectiveness of rural public health governance? To address these questions, this study focuses on the straw CS&T process and constructs a tripartite evolutionary game model involving governments, straw-specialized cooperatives, and farmers. Based on parameter calibration using real-world survey data, the study systematically examines the behavioral choices, dynamic evolutionary mechanisms, and stable equilibrium outcomes of multiple stakeholders under different policy scenarios. Particular attention is given to the effects of key variables, including government regulatory costs, subsidy incentives, cooperative operating returns, and farmer participation benefits, on the evolutionary trajectory of the system.

Compared with existing studies, the theoretical contributions of this study are reflected in three main aspects. First, this study moves beyond the conventional analytical framework that focuses solely on resource utilization efficiency. It formally incorporates rural public health risks into the research framework of straw governance and establishes an integrated perspective linking resource governance with public health governance. Second, unlike previous studies that mainly examine straw incorporation or industrial chain utilization, this study focuses on the straw CS&T process, a critical but long-neglected stage that largely determines the success of off-field straw utilization. It systematically reveals the dynamic strategic interactions and evolutionary patterns among governments, straw-specialized cooperatives, and farmers. Finally, this study adopts parameter settings and numerical simulations based on field survey data, which improves the consistency between the evolutionary game analysis and real-world policy scenarios. It also provides a more practically grounded analytical framework for behavioral decision-making research in agricultural waste governance. The findings offer theoretical support and policy references for optimizing straw off-field utilization incentives, improving the construction of the straw CS&T system, and strengthening source-oriented governance of rural public health risks. More importantly, the study helps promote a collaborative governance model characterized by effective government regulation, standardized cooperative operations, and active farmer participation. Such a governance framework can reduce open-field burning and extensive straw disposal practices at the source, while advancing green agricultural development, resource recycling, and rural public health governance in a coordinated manner.

## 2 Literature review

As the central hub of the entire straw utilization chain, the efficient and standardized operation of the straw CS&T system not only determines the efficiency of straw resource conversion and the realization of ecological and economic value (16, 20), but also directly affects environmental and health risks caused by practices such as open-field burning and random straw dumping, including air pollution, water contamination, and the breeding of disease vectors (13, 15). Current research on straw CS&T has developed into a multidimensional and multi-level research field, mainly focusing on cost control, influencing factors, and the optimization of organizational models (21). However, most existing studies regard straw governance primarily as an issue of resource utilization or environmental management. Limited attention has been given to the intrinsic relationship between straw CS&T and rural public health governance, and a systematic analytical framework that integrates both resource governance and public health objectives has yet to be established. Recent studies in environmental governance and sustainable resource management further suggest that the long-term effectiveness of governance systems depends not only on technological efficiency or economic returns, but also on institutional arrangements, public policy coordination, economic incentive mechanisms, and collaboration among multiple stakeholders (22–24). Through dynamic behavioral adjustment mechanisms, these factors influence the stability of resource governance systems, social welfare outcomes, and the sustainability of policy interventions. In the field of agricultural waste governance, the straw CS&T system also involves interest coordination and behavioral interactions among governments, straw-specialized cooperatives, and farmers. Its operational efficiency affects not only the effectiveness of resource recycling, but also rural environmental quality and public health security through behaviors such as straw burning and disorderly dumping. Therefore, it is necessary to formally incorporate public health risks into the research framework of straw CS&T governance from the perspective of multi-stakeholder dynamic collaboration.

In terms of research on the costs and influencing factors of straw CS&T, most scholars have relied on field survey data to analyze cost composition and key constraints from a full-chain perspective. Existing studies generally agree that collection, transportation, and loading-unloading costs constitute essential components of the straw CS&T system (25). Meanwhile, factors such as transportation distance, straw moisture content, storage losses, and the level of mechanization input significantly affect overall CS&T costs (26–28). When CS&T costs are excessively high, the sustainability of standardized straw disposal is often weakened. This may further lead to extensive disposal behaviors such as open-field burning and random dumping, thereby intensifying environmental pollution and health risk exposure in rural areas (29, 30). In addition, fragmented smallholder farming, difficulties in straw aggregation, and insufficient coverage of specialized service organizations further constrain the large-scale and stable operation of the CS&T system (16, 31). These studies provide important references for identifying operational bottlenecks in the straw CS&T system and for optimizing cost-sharing mechanisms. However, most of them still focus primarily on economic efficiency. The interaction between resource governance performance and public health risks has not been sufficiently explored.

In the field of straw CS&T model optimization, scholars have extensively explored diversified organizational models by considering regional agricultural production conditions, straw resource endowments, and industrial foundations. The main models commonly identified in China include the cooperative-led model, the enterprise-led model, and the government-guided model. Studies show that the cooperative-led model can effectively connect smallholder farmers with market actors, improving straw aggregation efficiency and service coverage (10). The enterprise-led model places greater emphasis on large-scale and industrialized operation, enhancing straw resource utilization efficiency through stronger integration of upstream and downstream value chains (32, 33). The government-guided model is mainly applied in regions with insufficient market participation, where fiscal support is used to ensure basic standardized disposal of straw (34). Existing research has primarily examined the operational efficiency, benefit distribution, and applicability conditions of different models, providing important references for optimizing regional straw CS&T systems. However, overall, these studies still focus more on resource utilization efficiency and economic feasibility. Systematic discussions on public health risk control outcomes and multi-stakeholder collaborative governance mechanisms under different governance models remain relatively insufficient.

The efficient operation of the straw CS&T system is essentially a dynamic process of behavioral adjustment and coordinated decision-making among multiple stakeholders, including governments, CS&T service organizations, and farmers, under conditions of bounded rationality. The strategic choices of different actors not only affect the operational efficiency of the CS&T system, but also influence rural environmental governance and public health risk levels through behaviors such as open-field burning and disorderly straw dumping (35, 36). Therefore, straw CS&T governance possesses a dual nature, encompassing both resource governance and public health governance, with its core lying in interest coordination and behavioral synergy among multiple stakeholders. Evolutionary game theory, which can effectively capture the processes of strategy learning and dynamic adjustment among bounded rational actors in long-term interactions, has been widely applied in recent years to studies on agricultural environmental governance and agricultural waste resource utilization (36). Existing research has mainly focused on behavioral interactions among governments, farmers, and enterprises. For instance, Jin et al. (37), from an evolutionary game perspective, analyzed the straw disposal decisions of farmers with differing preferences, revealing the interaction mechanisms between government policies and farmer strategy choices. Geng et al. (38) developed a three-party evolutionary game model involving farmers, enterprises, and local governments, finding that a higher degree of local government preference increased the likelihood of farmers and enterprises participating in straw return systems. Yang et al. (39) further pointed out that government subsidy intensity and regulatory strength significantly affect the willingness of enterprises and farmers to engage in straw resource utilization. These studies provide a methodological foundation for understanding stakeholder behavior in agricultural waste governance. However, their objectives are largely centered on improving economic returns and optimizing resource recycling efficiency. Public health risks are typically treated only as an indirect outcome of environmental governance and have not been formally incorporated into evolutionary game frameworks. In other words, existing studies generally lack an integrated analytical system that jointly considers public health risk constraints, multi-agent behavioral responses, and resource governance performance. From a practical governance perspective, long-term straw accumulation can become a major breeding ground for disease vectors such as rodents, mosquitoes, and flies, while open-field burning releases pollutants such as PM2.5, carbon monoxide (CO), and volatile organic compounds (VOCs), posing persistent threats to rural residents' respiratory health and living environment safety. Therefore, rural public health governance is not external to straw governance systems, but rather an embedded objective within multi-stakeholder behavioral interactions. By comparison, in fields such as construction waste management, some studies have begun to incorporate social welfare, environmental constraints, and health risks into evolutionary game frameworks. Liu and Teng (40) used an evolutionary game model to analyze decision-making interactions and stability strategies among multiple stakeholders in the construction waste recycling industry chain. Ma and Zhang (41) developed a dynamic evolutionary game model to examine the co-evolution of behaviors between construction enterprises and recycling firms under government incentives. Li et al. (42) found that contractors' environmental preferences significantly influence the proportion of illegal dumping and landfill disposal, which in turn affects environmental governance performance. Although these studies are not directly focused on straw governance, their approach of integrating non-economic constraints, social welfare objectives, and environmental health effects into evolutionary game analysis provides important theoretical references for constructing a tripartite evolutionary game model that jointly considers resource governance and public health objectives in this study.

A review of the existing literature indicates that research on straw CS&T has achieved substantial progress in areas such as cost control, organizational models, and stakeholder behavior analysis. However, several limitations remain. First, most studies focus on improving straw resource utilization efficiency or optimizing economic returns, while the intrinsic relationship between multi-stakeholder interactions in the CS&T process and public health risks has not been systematically examined from a public health governance perspective. Second, existing evolutionary game studies are mainly concentrated on straw incorporation and energy utilization processes. Relatively less attention has been paid to the CS&T system, which serves as a critical hub in the straw utilization chain. In particular, there is a lack of systematic research that formally incorporates public health risks into evolutionary game frameworks. Third, some studies still rely primarily on hypothetical parameters or literature-based assignments for numerical simulation. This limits their ability to accurately capture the behavioral characteristics of governments, straw-specialized cooperatives, and farmers in real-world contexts, leading to potential deviations between theoretical conclusions and practical governance scenarios (43, 44).

Based on this, the study is grounded in the central role and practical challenges of the straw CS&T system, and aligns with the modernization goals of rural public health governance. Focusing on three key stakeholders—government, straw-specialized cooperatives, and farmers—and drawing on extensive field surveys, expert interviews, and questionnaire data, this study constructs a tripartite evolutionary game model that integrates both resource utilization and public health governance objectives. Real-world survey data are introduced for parameter calibration and numerical simulation. The study systematically analyzes stakeholders' decision-making logic, interaction and evolutionary patterns, and key influencing factors. It also identifies the main conflicts of interest, core bottlenecks, and their constraints on rural public health governance performance within the CS&T system. Based on these findings, targeted and operational policy recommendations are proposed to promote the coordinated improvement of the CS&T system and rural public health governance efficiency. This work aims to provide theoretical support and practical guidance for addressing public health risks caused by extensive straw disposal and for advancing green agricultural development and rural revitalization strategies. The theoretical contributions of this study are mainly reflected in the following three aspects:

First, it constructs a tripartite evolutionary game model involving government, cooperatives, and farmers, systematically revealing the dynamic strategic interaction mechanism among multiple stakeholders. Existing studies are mostly based on two-player games or single-agent perspectives, which makes it difficult to capture the complex features of conflict and co-evolution among the three stakeholders across the full CS&T chain. This study breaks through these limitations by integrating government regulatory guidance, cooperative standardized operation, and farmer participation into a unified analytical framework. It dynamically simulates the strategic adjustment processes of the three parties under bounded rationality and identifies key variables and evolutionary pathways that affect system stability and equilibrium outcomes.

Second, it incorporates real-world survey data for parameter calibration, improving the practical relevance and policy applicability of the findings. Most existing evolutionary game studies in straw governance rely on hypothetical parameters or literature-based assignments, with limited ability to capture behavioral characteristics in real contexts. This study collects empirical data on behavioral preferences, cost-benefit structures, and risk perceptions of governments, cooperatives, and farmers in typical straw-producing regions of Northeast China through questionnaires, expert interviews, and field investigations. These data are used for model initialization and numerical simulation, making the results more consistent with real decision-making scenarios.

Third, it integrates dual objectives of resource utilization efficiency and rural public health governance by developing an evolutionary game model with embedded public health risk constraints. Unlike previous studies that primarily pursue straw resource utilization or economic efficiency maximization, this study introduces quantifiable health risk indicators and risk-control constraints into the payoff functions of all agents. It dynamically simulates how different policy combinations affect both public health outcomes and CS&T system efficiency, providing a theoretical basis for designing coordinated governance policies that balance resource recycling and health protection.

## 3 Model assumptions and construction

### 3.1 Model assumptions

**Figure 1:**
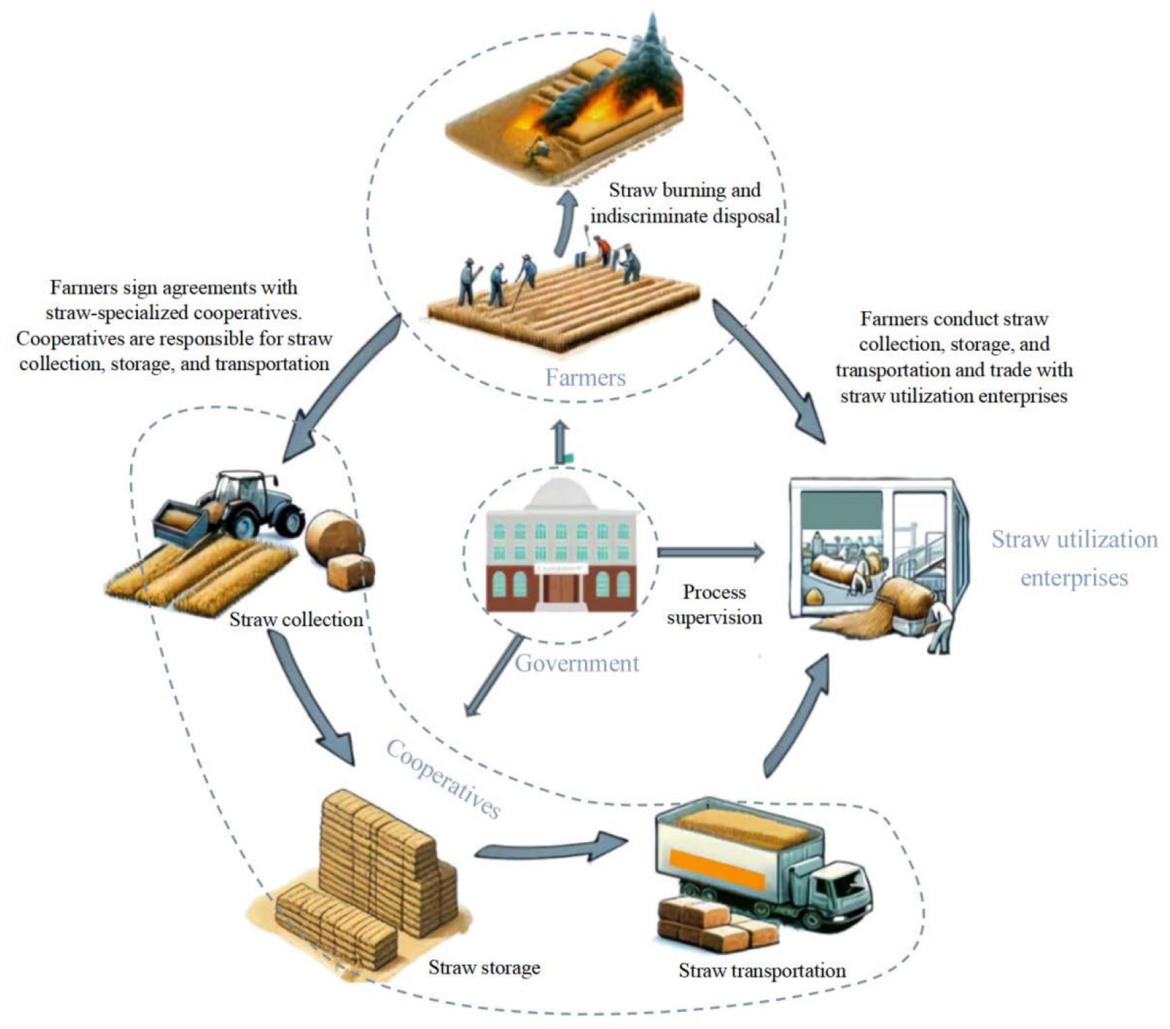
Relationship diagram of stakeholders in the straw CS&T process.

The successful advancement of straw removal from the fields relies on the collaborative interaction and close coordination of the three core stakeholders: the government, specialized straw cooperatives, and farmers. These three parties work together through multidimensional interactions to achieve efficient resource integration, precise information exchange, and reasonable benefit balancing. Their joint efforts drive the standardization and efficient operation of the entire straw CS&T process, laying a solid foundation for the scientific disposal of straw, preventing the chaotic and inefficient disposal practices at the source, and strengthening rural public health risk prevention. This study begins by systematically analyzing the behavioral decision characteristics and logical tendencies of the government, straw cooperatives, and farmers, clarifying the internal relationships, interactive mechanisms, and interest conflict dynamics between the stakeholders. The benefit relationships among the three parties are illustrated in Figure 1.

**Figure 2:**
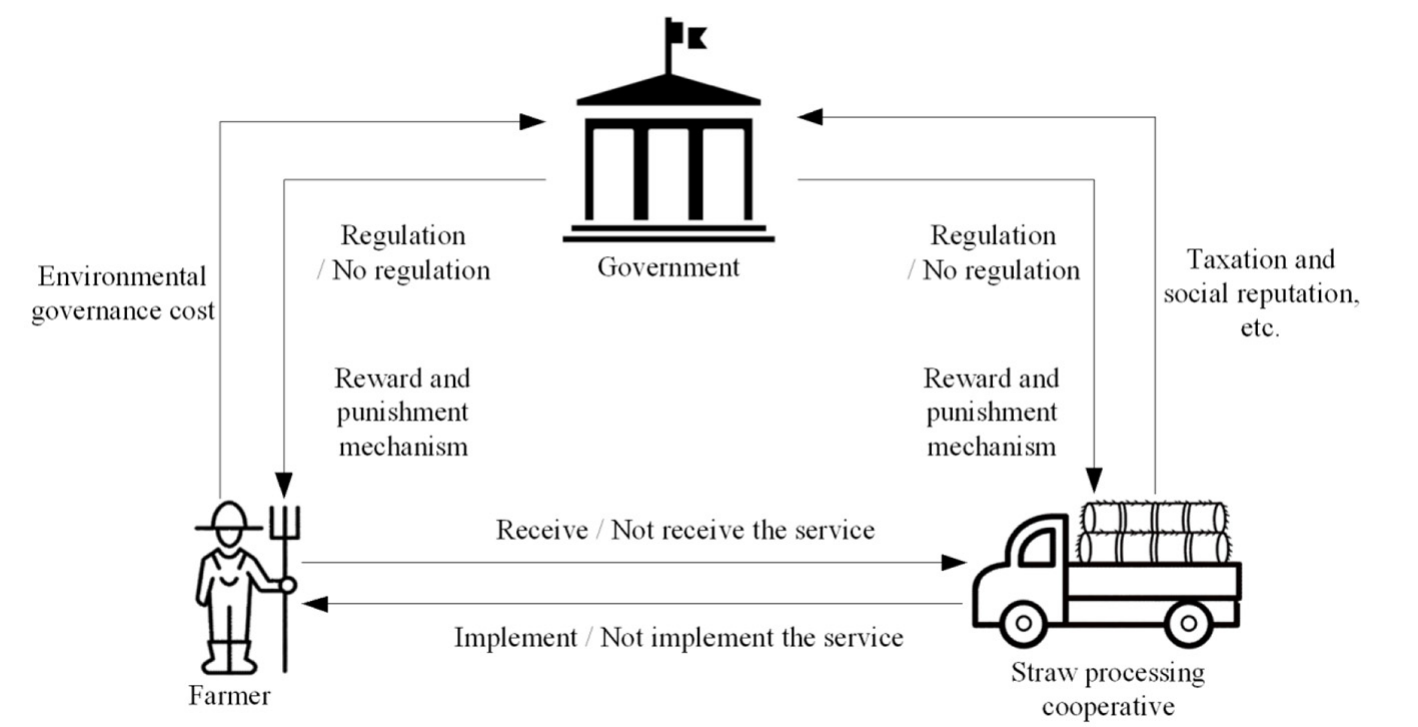
Conceptual diagram of the three-party evolutionary game model.

To construct a scientific tripartite evolutionary game model involving the government, straw-specialized cooperatives, and farmers, and to accurately capture the interaction logic among stakeholders in the straw CS&T process, this study aligns with the core objectives of rural public health governance, including source prevention and process control. The model is developed based on the practical characteristics of China's agricultural system, where smallholder farmers are widely dispersed in supply, cooperatives operate in a centralized manner, and the government plays a macro-regulatory role. It should be noted that, to ensure the empirical relevance and explanatory power of the model, key parameters such as subsidy levels, regulatory costs, and penalty standards are not set as purely theoretical assumptions. Instead, they are scientifically determined based on field survey data from 30 key counties for straw comprehensive utilization, as well as authoritative policy documents such as the Implementation Plan for Straw Comprehensive Utilization. Based on this, the conceptual structure of the tripartite evolutionary game model is shown in Figure 2, and the following assumptions are made.

**Hypothesis 1**. Definition of game participants and rationality characteristics. The government is defined as Player 1, straw-specialized cooperatives as Player 2, and farmers as Player 3. The three together constitute the core interest-related system of the straw CS&T process. All participants are assumed to be bounded rational agents whose strategic choices evolve and improve over time.

**Hypothesis 2**. Strategy sets and probability distributions of each player. The government's strategy set is {Regulate, Not Regulate}, where the probability of regulation is *x* and the probability of no regulation is 1 – *x*. The strategy set of straw-specialized cooperatives is {Provide CS&T Services, Not Provide CS&T Services}, with a probability *y* of providing services and a probability 1 – *y* of not providing them. The farmers' strategy set is {Accept Cooperative Service, Reject Cooperative Service}, where the probability of actively cooperating and accepting CS&T services is *z*, and the probability of rejecting cooperation and handling straw independently is 1 – *z*. All probability variables satisfy *x, y, z* ε [0, 1]. The strategy choices of the three stakeholders are assumed to be independent and dynamically adjusted over time.

**Hypothesis 3**. Government Payoff and Cost Functions and Their Associated Logic. When the government chooses to regulate, it incurs a regulatory cost *R*_1_. If cooperatives operate CS&T services normally, the government provides them with a basic operational subsidy *S*_1_, agricultural machinery purchase subsidy *S*_2_, straw treatment subsidy *S*_3_, tax incentives *S*_4_, and rewards *C*_1_ for exceeding performance targets. If the cooperative fails to provide services, a penalty *P*_1_ is imposed. For farmers, if they accept cooperative services, the government provides a dedicated subsidy *S*_5_; if they choose to burn straw, administrative fines *P*_2_ are imposed according to the Air Pollution Prevention and Control Law. Effective regulation also helps standardize straw disposal, reduce public health risks, and enhance public satisfaction, generating government credibility gains *G*_1_. Conversely, choosing not to regulate saves regulatory costs *R*_1_ but may damage government image, resulting in credibility losses *G*_2_. Additionally, farmers' open burning or indiscriminate disposal of straw can trigger negative public opinion, increase public health governance pressure, and generate adverse effects *B*_1_, while causing pollution of air, water, and soil, and fostering vector-borne hazards *W*.

**Hypothesis 4**. Cooperative Payoff and Cost Functions and Their Associated Logic. When the cooperative chooses to implement services, it bears direct processing costs *R*_2_ (including field CS&T), indirect costs *R*_3_ (equipment acquisition, technology development, staff training), and payments to farmers as straw collection subsidies *S*_6_. The cooperative generates market revenue *L*_1_ from selling straw to downstream enterprises. If the government enforces regulation, compliant cooperatives are eligible for the basic operational subsidy *S*_1_, machinery purchase subsidy *S*_2_, straw treatment subsidy *S*_3_, tax incentives *S*_4_, and performance reward *C*_1_. If the cooperative chooses not to provide services and is subject to government regulation, it must pay the penalty *P*_1_ and forfeits any market revenue and policy subsidies.

**Hypothesis 5**. Farmer Payoff and Cost Functions and Their Associated Logic. When farmers choose to accept cooperative services, they transfer straw to the cooperative and receive both the cooperative's straw collection subsidy *S*_6_ and the government's dedicated subsidy *S*_5_, while incurring necessary time and labor costs *R*_4_. Under this scenario, farmers effectively avoid administrative penalties and environmental health losses. When farmers choose self-disposal (i.e., straw burning), the residual straw fertility partially substitutes chemical fertilizers, generating fertilizer savings *L*_2_. Under government regulation, open burning incurs an administrative fine *P*_2_. Moreover, the resulting air pollution significantly increases farmers' risk of respiratory diseases, generating implicit personal health costs *H*, and degrades the rural living environment, directly threatening both farmers' quality of life and rural public health safety.

### 3.2 Model construction and payoff matrix

Based on the above assumptions and parameter settings, a three-player evolutionary game matrix is constructed with the government, cooperatives, and farmers as the core game participants, where the net payoffs under different strategy combinations serve as the key analytical representation. In the matrix, the payoff vector corresponding to each strategy combination is ordered as government net payoff, cooperative net payoff, and farmer net payoff, respectively. Each payoff component is strictly derived from the cost–benefit function logic established in the preceding sections and comprehensively incorporates policy subsidies, operating costs, market revenues, rewards and penalties, as well as public health–related gains and losses. In this way, the matrix accurately characterizes the payoff equilibrium logic underlying strategic interactions among the three parties. The specific evolutionary game matrix is presented in Table 1.

**Table 1:** Payoff matrix of the tripartite evolutionary game.

Strategy combinations	Government payoff	Straw specialized cooperative payoff	Farmer payoff
Supervise, implement, accept	–*R*_1_ – *S*_1_ – *S*_2_ – *S*_3_ – *S*_4_ – *C*_1_ + *G*_1_	–*R*_2_ – *R*_3_ + *L*_1_ + *S*_1_ + *S*_2_ + *S*_3_ + *S*_4_ + *C*_1_ – *S*_6_	*S*_6_ + *S*_5_ – *R*_4_
Supervise, implement, not accept	–*R*_1_ – *S*_1_ – *S*_2_ – *S*_3_ – *S*_4_ – *B*_1_ – *W* + *G*_1_ + *P*_2_	–*R*_3_ + *S*_1_ + *S*_2_ + *S*_3_ + *S*_4_	*L*_2_ – *P*_2_ – *H*
Supervise, not implement, accept	–*R*_1_ – *S*_5_ + *P*_1_ + *G*_1_	–*P*_1_	*S*_5_ – *R*_4_
Supervise, not implement, not accept	–*R*_1_ + *P*_1_ – *B*_1_ – *W* + *G*_1_ + *P*_2_	–*P*_1_	*L*_2_ – *P*_2_ – *H*
Not supervise, implement, accept	–*G*_2_	*L*_1_ – *R*_2_ – *R*_3_ – *S*_6_	*S*_6_
Not supervise, implement, not accept	–*G*_2_ – *B*_1_ – *W*	–*R*_3_	*L*_2_ – *H*
Not supervise, not implement, accept	–*G*_2_	0	0
Not supervise, not implement, not accept	–*G*_2_ – *B*_1_ – *W*	0	*L*_2_ – *H*

## 4 Equilibrium analysis of the three-party evolutionary game model

### 4.1 Dynamic equation analysis of government replication

The expected benefits of “supervise” and “not supervise” decisions of the government are *V*_11_, *V*_11_, and the average benefit *V*_1_, respectively:


$$\begin{array}{l} V_{11\;}=\;yz\left(-R_{1}-S_{1}-S_{2}-S_{3}-S_{4}-C_{1}\;+\;G_{1}\right)\\ \;+\;y\left(1-z\right)\left(-R_{1}-S_{1}-S_{2}-S_{3}-S_{4}-B_{1}-W\;+\;G_{1\;}+\;P_{2}\right)\\ \;+\left(1\;-\;y\right)z\left(-R_{1}-S_{5}\;+\;P_{1\;}+\;G_{1}\right)\;+\;\left(1-y\right)\left(1-z\right)\\ \;\times \left(-R_{1\;}+\;P_{1}-B_{1}-W\;+\;G_{1\;}+\;P_{2}\right) \end{array}$$ (1)

$$\begin{array}{l} V_{12}\;=\;yz\left(-G_{2}\right)+y\left(1-z\right)\left(-G_{2}-B_{1}-W\right)+\left(1-y\right)z\left(-G_{2}\right)\\ \;+\left(1-y\right)\left(1-z\right)\left(-G_{2}-B_{1}-W\right) \end{array}$$ (2)

$$\begin{array}{l} {\bar{V}}_{1}\;=\;xV_{11\;}+\;\left(1-x\right)V_{12} \end{array}$$ (3)

The replication dynamic equation of government is obtained by:


$$\begin{array}{l} F(x)=x(1-x)[G_{1}+G_{2}-R_{1}+P_{1}+P_{2}-z(P_{2}+S_{5})\\ \;-y(P_{1}+S_{1}+S_{2}+S_{3}+S_{4}+z(C_{1}-S_{5}))] \end{array}$$ (4)

From Equation 4, when $y\;=\;\frac{G_{1}+G_{2}-R_{1\;}+\;P_{1}+P_{2}-z\left(P_{2\;}+\;S_{5}\right)}{P_{1}\;+{\;S}_{1}\;+{\;S}_{2\;}+\;S_{3\;}+\;S_{4}+z\left(C_{1}-S_{5}\right)}=y^{*}$, *F*(*x*)≡0. At this point, the government's strategy for any value of *x* is in a steady state. This implies that the government is in a steady state whether or not it adopts a regulatory strategy. When *y*≠y^*^, making *F*(*x*) = 0 yields *x* = 0, *x* = 1, which may be the evolutionary stability point. From the stability theorem of the replicated dynamic equation, it is known that *x* as a stabilizing strategy needs to comply with *F*(*x*) = 0 and *F*′(*x*) < 0.

The derivative of the replicated dynamic equations is obtained:


$$\begin{array}{l} F^{\prime }(x)=(2x-1)[G_{1}+G_{2}-R_{1}+P_{1}+P_{2}-z(P_{2}+S_{5})\\ \;-y(P_{1}+S_{1}+S_{2}+S_{3}+S_{4}+z(C_{1}-S_{5}))] \end{array}$$ (5)

From Equation 5, when *y*<y*, $F^{\prime }{\left(x\right)}_{|x=0}>0$, $F^{\prime }{\left(x\right)}_{|x=1}<0$. At this time, the evolutionary stabilization point and stabilization strategy of the government is to choose to regulate. On the contrary, when *y*>y*, $F^{\prime }{\left(x\right)}_{|x=0}<0$, $F^{\prime }{\left(x\right)}_{|x=1}>0$. At this time, the point of evolutionary stability is that the government's stabilization strategy is not to regulate.

### 4.2 Equation analysis of replication dynamics of straw professional cooperatives

The expected benefits of “implement” and “not implement” decisions of the straw professional cooperatives are *V*_21_, *V*_22_, and the average benefit *V*_2_, respectively:


$$\begin{array}{l} V_{21}\;=xz\left(L_{1}-R_{2}-R_{3}-S_{6}+S_{1\;}+\;S_{2}\;+{\;S}_{3}+S_{4\;}+\;C_{1}\right)\\ \;+x\left(1-z\right)\left(-R_{3\;}+\;S_{1\;}+\;S_{2}+S_{3\;}+\;S_{4}\right)\\ \;+\left(1-x\right)z\left(L_{1}-R_{2}-R_{3}-S_{6}\right)+\left(1-x\right)\left(1-z\right)\left(-R_{3}\right) \end{array}$$ (6)

$$\begin{array}{l} V_{22}\;=xz\left(-P_{1}\right)+x\left(1-z\right)\left(-P_{1}\right)+\left(1-x\right)z\left(0\right)\\ \;+\left(1-x\right)\left(1-z\right)\left(0\right)\;=\;-xP_{1} \end{array}$$ (7)

$$\begin{array}{l} {\bar{V}}_{2}\;=\;yV_{21}+\left(1-y\right)V_{22} \end{array}$$ (8)

The replication dynamic equation of straw professional cooperatives is obtained by:


$$\begin{array}{l} F(y)=y(1-y)[z(L_{1}-R_{2}-S_{6})-R_{3}+x(S_{1}+S_{2}+S_{3}\\ \;+\;S_{4}+P_{1})+xzC_{1}] \end{array}$$ (9)

From Equation 9, when $x\;=\;\frac{R_{3}-z\left(L_{1}-R_{2}-S_{6}\right)}{S_{1\;}+\;S_{2\;}+\;S_{3\;}+{\;S}_{4\;}+\;P_{1\;}+\;zC_{1}}=x^{*}$, *F*(*y*)≡0. At this point, *y* takes any value, and the strategy of the straw professional cooperatives is in a steady state. When *z*≠z*, making *F*(y) = 0 yields *y* = 0, *y* = 1, which may be the evolutionary stability point. From the stability theorem of the replicated dynamic equation, it is known that *y* as a stabilizing strategy needs to comply with *F*(*y*) = 0 and *F*′(*y*) < 0.

The derivative of the replicated dynamic equations is obtained:


$$\begin{array}{l} F^{\prime }(y)=(2y-1)[z(L_{1}-R_{2}-S_{6})-R_{3}+x(S_{1}+S_{2}+S_{3}\\ \;+\;S_{4}+P_{1})+xzC_{1}] \end{array}$$ (10)

From Equation 10, when *z*<z^*^, $F^{\prime }\left(y\right){|}_{y=0}<0$, $F^{\prime }\left(y\right){|}_{y=1}>0$. At this time, *y* = 0 is the point of evolutionary stabilization, and the stabilization strategy of straw professional cooperatives is not to carry out the straw CS&T. On the contrary, when *z* > z^*^, $F^{\prime }\left(x\right){|}_{y=0}>0$, $F^{\prime }\left(x\right){|}_{y=1}<0$. At this time, *y* = 1 is the evolutionary stability point, and the stabilization strategy of the straw professional cooperatives is to carry out straw CS&T.

### 4.3 Equation analysis of farmers' replication dynamics

The expected benefits of “accept” and “not accept” decisions of the farmers are *V*_31_, *V*_32_, and the average benefit *V*_3_, respectively:


$$\begin{array}{l} V_{31}\;=xy\left(S_{6}+S_{5}-R_{4}\right)+x\left(1-y\right)\left(S_{5}-R_{4}\right)\\ \;+\left(1-x\right)y\left(S_{6}\right)-\left(1-x\right)\left(1-y\right)\left(0\right) \end{array}$$ (11)

$$\begin{array}{l} V_{32}\;=xy\left(L_{2}-P_{2}-H\right)+x\left(1-y\right)\left(L_{2}-P_{2}-H\right)\\ \;+\left(1-x\right)y\left(L_{2}-H\right)+\left(1-x\right)\left(1-y\right)\left(L_{2}-H\right) \end{array}$$ (12)

$$\begin{array}{l} {\bar{V}}_{3}\;=\;zV_{31}+\left(1-z\right)V_{32} \end{array}$$ (13)

The replication dynamic equation of farmers is obtained by:


$$\begin{array}{l} F\left(z\right)\;=\;z\left(1-z\right)\left[yS_{6}+x\left(S_{5\;}+\;P_{2}-R_{4}\right)-L_{2}+H\right] \end{array}$$ (14)

From Equation 14, when $y\;=\;\frac{L_{2}-H-x\left(S_{5}+P_{2}-R_{4}\right)}{S_{6}}=y^{*}$, *F*(*z*)≡0. At this point, *z* takes any value, and the strategy of the farmers is in a steady state. When *y*≠y*, making *F*(*z*) = 0 yields *z* = 0, *z* = 1, which may be the evolutionary stability point. From the stability theorem of the replicated dynamic equation, it is known that *z* is a stabilizing strategy that needs to comply with *F*(*z*) = 0 and *F*′(*z*) < 0.

The derivative of the replicated dynamic equations is obtained:


$$\begin{array}{l} F^{\prime }\left(z\right)\;=\;\left(2z-1\right)\left[yS_{6}+x\left(S_{5\;}+\;P_{2}-R_{4}\right)-L_{2}+H\right] \end{array}$$ (15)

From Equation 15, when *y*<y^*^, $F^{\prime }\left(z\right){|}_{z=0}<0$, $F^{\prime }\left(z\right){|}_{z=1}>0$. At this time, the point of evolutionary stabilization and the farmer's stabilization strategy is not to sell the straws. On the contrary, when *y* > y*, $\;F^{\prime }\left(z\right){|}_{z=0}>0$, $F^{\prime }\left(z\right){|}_{z=1}<0.$ At this time, *z* = 1 is the evolutionary stability point, and the farmer's stabilization strategy is to sell the straws.

### 4.4 Evolutionary stable equilibrium analysis

From Equations 4, 9, and14, the system of equations for the replication dynamics of government-straw specialized cooperatives-farmers is given as:


$$\{\begin{array}{lll} F(x)\; & = & x(1-x)[G_{1}+G_{2}-R_{1\;}+\;P_{1\;}+\;P_{2}-z(P_{2}+S_{5})\\ \; & - & \;y(P_{1}+S_{1\;}+\;S_{2}+S_{3\;}+\;\;S_{4}+z(C_{1}-S_{5}))]\\ F(y)\; & = & y(1-y)[z(L_{1}-R_{2}-S_{6})-R_{3}+x(S_{1}+S_{2\;}+\;S_{3\;}\\ \; & + & \;S_{4\;}+P_{1})+xzC_{1}]\\ F(z)\; & = & z(1-z)[yS_{6}+x(S_{5\;}+\;P_{2}-R_{4})-L_{2}+H] \end{array}$$

The system of joint equations, when *F*(*x*) = *F*(*y*) = *F*(*z*) = 0, we can get nine equilibrium points which are (0, 0, 0), (0, 1, 0), (0, 0, 1), (0, 1, 1), (1, 1, 0), (1, 1, 0), (1, 0, 1), (1, 1, 1), (x^*^, y^*^, z^*^). Since (x^*^, y^*^, z^*^) is a non-asymptotic steady state, only the first eight special equilibrium points are discussed in this paper, and the Jacobian matrix replicating the dynamic equation is shown in Equation 17.


$$\begin{array}{l} J=\left[\begin{array}{c} \begin{array}{ccc} J_{11} & J_{12} & J_{13}\\ J_{21} & J_{22} & J_{23}\\ J_{31} & J_{32} & J_{33} \end{array} \end{array}\right]=\left[\begin{array}{ccc} \partial F\left(x\right)/\partial x & \partial F\left(x\right)/\partial y & \partial F\left(x\right)/\partial z\\ \partial F\left(y\right)/\partial x & \partial F\left(y\right)/\partial y & \partial F\left(y\right)/\partial z\\ \partial F\left(z\right)/\partial x & \partial F\left(z\right)/\partial y & \partial F\left(z\right)/\partial z \end{array}\right] \end{array}$$ (16)

Among them.


$$\{\begin{array}{lll} J_{11}\; & = & \left(1-2x)[G_{1\;}+\;G_{2}-R_{1\;}+\;P_{1}+P_{2}-z(P_{2}+S_{5}\right)\\ \; & - & \;y(P_{1}+S_{1}+\;S_{2}+S_{3\;}+\;S_{4}+\;z(C_{1}-S_{5}))]\\ J_{12}\; & = & -x(1-x)[P_{1}+S_{1}+S_{2}+S_{3}+S_{4}+z(C_{1}-S_{5})]\\ J_{13}\; & = & \;-x(1-x)[P_{2}+S_{5}+y(C_{1}-S_{5})]\\ J_{21}\; & = & \;y(1-y)[S_{1}+S_{2}+S_{3}+S_{4}+P_{1}+zC_{1}]\\ J_{22}\; & = & \;(1-2y)[z(L_{1}-R_{2}-S_{6})-R_{3}+x(S_{1}+S_{2}\\ \; & + & \;S_{3}+S_{4\;}+\;P_{1})+xzC_{1}]\\ J_{23}\; & = & \;y(1-y)[L_{1}-R_{2}-S_{6}+xC_{1}]\\ J_{31}\; & = & \;z(1-z)[S_{5\;}+\;P_{2}-R_{4}]\\ J_{32}\; & = & \;z(1-z)[S_{6}]\\ J_{33}\; & = & \;(1-2z)[yS_{6\;}+\;x(S_{5}+P_{2}-R_{4})-L_{2}+H] \end{array}$$

Based on the equilibrium point stability test proposed by Friedman in 1998, the stability of each equilibrium point can be judged based on the positivity and negativity of the matrix determinant values and traces. Specifically, is an ESS only if makes all the eigenvalues of the Jacobi matrix negative at the same time. Accordingly, the evolutionary stabilization strategies of the government, the straw professional cooperatives, and the farmers are solved. The above eight equilibrium points are substituted into this Jacobi matrix, and the corresponding eigenvalues are obtained, respectively, as shown in Table 2.

**Table 2:** Eigenvalues of equilibrium points.

Equilibrium points	λ_1_	λ_2_	λ_3_	Notation	Results	Stable conditions
*X*_1_(0, 0, 0)	*G*_1_ + *G*_2_ − *R*_1_ + *P*_1_+*P*_2_	−*R*_3_	*H* − *L*_2_	+ – ×	Instability point	
*X*_2_(1, 0, 0)	− (*G*_1_ + *G*_2_ − *R*_1_ + *P*_1_+*P*_2_)	*S*_1_ + *S*_2_ + *S*_3_ + *S*_4_ + *P*_1_ − *R*_3_	*S*_5_+*P*_2_ − *R*_4_ − *L*_2_+*H*	×+ ×	Instability point	
*X*_3_(0, 1, 0)	*G*_1_+ *G*_2_ − *R*_1_ + *P*_2_ − (*S*_1_ + *S*_2_+*S*_3_+*S*_4_)	*R*_3_	*S*_6_ − *L*_2_+*H*	×+ ×	Instability point	
*X*_4_(0, 0, 1)	*G*_1_ + *G*_2_ − *R*_1_ + *P*_1_ − *S*_5_	*L*_1_−*R*_2_−*R*_3_−*S*_6_	*L*_2_ − *H*	× – ×	ESS	
*X*_5_(1, 1, 0)	− (*G*_1_+*G*_2_ − *R*_1_ + *P*_2_ − *S*_1_ − *S*_2_ − *S*_3_ − *S*_4_)	− (*S*_1_+*S*_2_+*S*_3_+*S*_4_+*P*_1_ − *R*_3_)	*S*_6_+*S*_5_+*P*_2_ − *R*_4_ − *L*_2_+*H*	+ × +	Instability point	
*X*_6_(1, 0, 1)	− (*G*_1_+*G*_2_ − *R*_1_+*P*_1_ − *S*_5_)	*L*_1_ − *R*_2_ − *S*_6_ − *R*_3_+*S*_1_+*S*_2_+*S*_3_+*S*_4_+*P*_1_+*C*_1_	− (*S*_5_+*P*_2_ − *R*_4_ − *L*_2_+*H*)	× × –	ESS	
*X*_7_(0, 1, 1)	*G*_1_+*G*_2_ − *R*_1_ − *S*_1_ − *S*_2_ − *S*_3_ − *S*_4_ − *C*_1_	− (*L*_1_ − *R*_2_ − *S*_6_ − *R*_3_)	− (*S*_6_ − *L*_2_+*H*)	× × –	ESS	
*X*_8_(1, 1, 1)	−(*G*_1_+ *G*_2_ − *R*_1_− *S*_1_ − *S*_2_ − *S*_3_ − *S*_4_ − *C*_1_)	−(*L*_1_ − *R*_2_ − *S*_6_ − *R*_3_ + *S*_1_ + *S*_2_ + *S*_3_+ *S*_4_+ *P*_1_ + *C*_1_)	− (*S*_6_ + *S*_5_ + *P*_2_ − *R*_4_ − *L*_2_ + *H*)	× – –	ESS	

× indicates an unknown sign. ESS stands for evolutionarily stable strategy.

*G*_1_ + *G*_2_ − *R*_1_ + *P*_1_ − *S*_5_ < 0, *L*_2_ − *H* < 0.

*G*_1_ + *G*_2_ − *R*_1_ + *P*_1_ − *S*_5_ > 0, *L*_1_ − *R*_2_ − *S*_6_ − *R*_3_ + *S*_1_ + *S*_2_ + *S*_3_ + *S*_4_+ *P*_1_+ *C*_1_ < 0.

*G*_1_ + *G*_2_ − *R*_1_ − *S*_1_ − *S*_2_ − *S*_3_ − *S*_4_ − *C*_1_ < 0, *L*_1_ − *R*_2_ − *S*_6_ − *R*_3_ > 0.

*G*_1_ + *G*_2_ − *R*_1_ − *S*_1_ − *S*_2_ − *S*_3_ − *S*_4_ − *C*_1_ > 0.

During the dynamic evolutionary game among the government, CS&T cooperatives, and farmers, if the corresponding stability conditions are satisfied, four stable points can emerge:

Scenario 1: Under Condition 1, the system converges to *X*_4_(0, 0, 1), representing a non-ideal state of “no government regulation, no cooperative participation, and farmers accepting services.” In this case, insufficient regulatory benefits lead to government inaction, while cooperatives refuse to provide CS&T services. However, because the hidden health costs of straw burning are higher than the savings from chemical fertilizers, farmers exhibit a strong willingness for off-field straw removal and environmental protection. Yet, due to the lack of available services, the governance process becomes stagnant.

Scenario 2: Under Condition 2, the system converges to *X*_6_(1, 0, 1). Even though the government chooses to regulate and farmers are willing to cooperate, the CS&T system still fails to establish when cooperative operating costs are excessively high, resulting in negative returns. Field surveys show that many straw collection intermediaries in Heilongjiang Province have been forced to exit the market or fall into operational difficulties due to high collection costs and limited profit margins. This indicates that administrative regulation alone, without economic feasibility, is insufficient to eliminate pollution risks at the source.

Scenario 3: Under Condition 3, the system converges to *X*_7_(0, 1, 1). In this case, cooperatives and farmers have formed a positive interaction, but due to insufficient government regulatory benefits, the government chooses not to regulate. Although basic straw handling can be maintained, the absence of macro-level regulation and baseline monitoring makes the rural public health protection system highly vulnerable to collapse under market fluctuations.

Scenario 4: Under Condition 4, the system converges to *X*_8_(1, 1, 1), where the combined gains from government credibility exceed the costs of regulation and subsidies, and the system reaches an ideal state. Field investigations show that the Denengda Cooperative in Xiaowuzhan Town, Boli County, Heilongjiang Province has established an operational model of “unified dispatching, zonal responsibility, and door-to-door collection.” In Hulin City, under government guidance, a straw CS&T network characterized by “enterprise + cooperative + farmer” has been formed. Farmers increase their annual income by more than 5,000 yuan per household through straw sales. In this scenario, the straw CS&T system operates efficiently, effectively preventing pollution from open-field burning at the source and fully achieving rural public health governance objectives.

## 5 Numerical experiments and simulation

### 5.1 Initial parameter settings

All parameters in this study are calibrated primarily based on field survey data from 30 nationally designated key counties for comprehensive straw utilization, ensuring both authority and comprehensiveness of the data sources. Baseline data are mainly drawn from current policy documents on straw utilization, the China Agricultural Statistical Yearbook, the China Land and Resources Statistical Yearbook, and other official publications, supplemented and cross-validated with publicly available information from news reports. For several latent parameters in the model that are difficult to quantify directly, scientific estimation and calibration were conducted through multi-regional field investigations and expert consultations within the industry, ensuring a high degree of consistency between parameter values and real-world straw CS&T practices. The specific parameter values used in the game system are reported in Table 3, providing a solid and reliable empirical foundation for subsequent model derivation, evolutionary stability analysis, and numerical simulation.

**Table 3:** Initial assignment of each variable parameter based on real data.

Parameters	Parameter meaning	Data source	Numerical value (hundreds of millions of RMB)
*R*_1_	Costs of government regulation	According to the 2023 Heilongjiang Provincial People's Government General Office issued the “Heilongjiang Provincial straw comprehensive utilization work implementation plan (Interim),” the key counties should be required to carry out the comprehensive utilization of straw demonstration base construction, monitoring and evaluation of straw returned to the field, monitoring of the crop-grass-grain ratio and straw collection coefficient of the special tasks, each key county subsidies of 450,000 yuan	0.14
		45 × 30 = 1,350 w	
*S*_1_	The government gives basic subsidies to straw treatment straw professional cooperatives.	According to the “Heilongjiang Provincial straw comprehensive utilization work implementation plan (provisional),” each key county can be in the centrally issued straw comprehensive utilization of the pilot county funds, a separate 3 million yuan, straw processing and utilization of the main body, straw collection, storage and transportation system and other aspects of straw off the field to support. The utilization of straw off the field in Heilongjiang Province accounts for about 27% of the comprehensive utilization of straw	0.24
		300 × 30 × 0.27 = 24.30 million	
*S*_2_	The government grants agricultural machinery purchase subsidies to straw treatment straw professional cooperatives.	Qitaihe City, agricultural machinery purchase subsidies applied for a total of 2,974, benefiting 2,379 households, enjoying subsidies for 3,021 sets of agricultural machinery, with a total subsidy of 50,172,500 yuan. Qitaihe cultivated area of 1.157 million mu, per mu subsidy of 43.6 yuan. Thirty key counties and districts cultivated an area of about 54.68 million mu. In addition, experts said the value of straw off-the-field machinery accounted for about 7% of all machinery	1.67
		43.6 × 5,468 × 0.07 = 166.88 million	
*S*_3_	Government subsidies for straw treatment given to straw professional cooperatives	According to the notice of the General Office of the Heilongjiang Provincial People's Government on the issuance of the Implementation Plan for Comprehensive Utilization of Straw in Heilongjiang Province (provisional), the utilization of straw away from the field, according to the actual amount of corn and rice straw utilized in the year, the provincial government will give a subsidy of 20 yuan per ton, and the upper limit of subsidy for a single project will not exceed 4 million yuan. Heilongjiang province's comprehensive utilization of straw amounts to 83.647 million tons, 30 key counties and districts cultivation area of about 54.68 million mu, and Heilongjiang province's sowing area of 228.6158 million mu. The utilization of straw off the field in Heilongjiang province accounts for about 27% of the comprehensive utilization of straw	1.08
		8,364.7 × (5,468/22,861.58) × 27% × 20 = 108 million	
S_4_	The government grants tax incentives to straw professional cooperatives.	The State Administration of Taxation (SAT) promulgated the circular on issuing the scope of primary processing of agricultural products enjoying preferential policies on enterprise income tax (for trial implementation), stating that in terms of value-added tax (VAT), the policy of 50% instant VAT refund (at a rate of 13%) shall be implemented for the sales of qualified straw pulp and other comprehensive utilization products produced by taxpayers using agricultural straws themselves. Baoji City Union's straw processing professional cooperatives have an annual processing and storage capacity of more than 50,000 tons of straw and a profit of more than 1.8 million yuan. The amount of straw leaving the field in 30 key counties in Heilongjiang is 8364.7^*^(5468/22861.58)^*^27%=5.4 million tons	
		180/5^*^540^*^0.13^*^0.5=1263.6w	0.13
S_5_	Government subsidies to farmers	According to the “Comprehensive utilization of straw in Heilongjiang Province (Interim),” the use of straw away from the field, according to the actual amount of corn and rice straw used in the year, the provincial government will give a subsidy of 20 yuan per tonne, and the upper limit of subsidies for a single project will not exceed 4 million yuan	1.08
		8,364.7 × (5,468/22,861.58) × 27% × 20 = 108 million	
*S*_6_	Subsidies to farmers from straw professional cooperatives	Jiangsu Province Dongxin Farm signed a contract with the straw purchase straw professional cooperatives, the straw professional cooperatives to the farm 75/ha (1 ha = 15 acres), that is, 5 yuan/mu	0.74
		5,468 × 5 × 0.27 = 73.82 million	
*C*_1_	The government grants bonuses to straw professional cooperatives	According to the table of straw collection and utilization volume and allocation of award funds for straw collection and utilization enterprises (sites) in Jiangyan District in 2022, the award will be RMB 3.11 per ton	0.17
		540 × 3.11 = 16.79 million	
*P*_1_	Government fines for straw professional cooperatives that do not provide services	According to the “Heilongjiang Province straw comprehensive utilization work implementation plan (provisional),” can not be quality and quantity to complete the target task, to inform the criticism, not complete the straw comprehensive utilization rate of the established tasks and objectives of the deduction of subsidies, straw comprehensive utilization rate than the established target of each one percentage point lower, to the county (city, district) as a unit to deduction of the annual straw comprehensive utilization of funds 1 million yuan	0.3
		100 × 30 = 30 million	
*P*_2_	Government fines for farmers for straw burning and dumping	Since the work to effectively address the open burning of straw in 2022–2023, a total of 136 fire points for open burning of straw and root stubble residues have been identified in Heilongjiang Province. According to the relevant provisions of the Air Pollution Prevention and Control Law, the pollution caused by open straw burning can be penalized from 500 yuan to 2,000 yuan, taking a median of 1,250 yuan	0.0017
		136 × 1,250 = 0.17 million	
*R*_2_	Other costs of straw collection, storage and transportation by straw professional cooperatives (introduction of new equipment, technology, etc.)	Qiqihar Xinxing modern agricultural agricultural machinery professional cooperative has fixed assets of 50 million yuan and an operating capacity of 150,000 standard mu (1 standard mu = 1.6 mu). The value of straw leaving the field machinery accounts for about 7% of all machinery.	2.2
		5,000/(15 × 1.6) × 0.07 × 5,468 × 0.27 = 215.30 million	
*R*_3_	Basic costs of straw collection, storage and transportation by straw professional cooperatives	The average cost per ton of straw is RMB 264.4 when the straw recycling company adopts the mechanical model.	14.3
		540 × 264.4 = 1,427.76 million	
*R*_4_	Farmer time and labor costs	When farmers cooperate with straw-specialized cooperatives in off-field straw operations—such as initial field residue cleaning, centralized collection and preprocessing, coordination of mechanized operations, and loading-unloading assistance—they are required to invest a certain amount of time and labor. Considering the recent outflow of rural labor and the significant increase in labor costs, and based on field surveys of typical farms as well as existing literature on the opportunity cost of rural labor, the comprehensive labor and time cost per ton of straw is set at 380 yuan	20.5
		Calculation result: 540 × 385.4 = 205,200 w	
*L*_1_	Proceeds from the sale of straw	The total amount of straw comprehensively utilized in a certain province is 83.647 million tons. The cultivated area of 30 key counties and districts is approximately 54.68 million mu, while the total sown area of the province is 228.6158 million mu. According to market quotations obtained through field visits to biomass power plants, paper mills, and feed enterprises, the current average transaction price for straw as a resource factor is stable at around 400 yuan per ton	21.6
		Calculation result: 83.647 × (54.68/228.6158) × 0.27 × 400 = 216,058 w	
*B*_1_	Government negative externality	Referring to the parameter settings in Zhou et al. (48) for environmental pollution control game models, this study sets *B*_1_ slightly above the basic regulatory cost to reflect the rigid constraint of government efforts in pollution management	0.41
*H*	Implicit personal health cost	Based primarily on Huang et al. (49) and Deuja et al. (29), which assess respiratory health risks from straw burning, and incorporating Luo et al. (12) regional air pollution monitoring data, this value is converted into the expected medical expenses and labor capacity losses incurred by farmers due to illness. The implicit personal health cost is calculated comprehensively from expected medical expenditures, labor time loss, and life-health depreciation resulting from exposure to straw burning smoke. According to this assessment, the implicit personal health cost for farmers in 30 key counties due to exposure to straw burning is approximately 15 million RMB	0.15
*W*	Public health hazard loss	Following the logic in Xu et al. (50) regarding coordinated governance of agricultural non-point source pollution, when farmers adopt non-green production practices such as straw burning, significant social welfare losses occur. In this study, this is quantified as public health hazard loss, with a value set at 0.8	0.8
*G*_1_	Government credibility gain	Based on Chen et al. (44), who examined collaborative governance of public crises in China, government decisions are influenced not only by economic costs but also by perceived reputational gains and losses. *G*_1_ represents the social credibility premium obtained by the government through actively fulfilling regulatory responsibilities. Considering the context of this study, *G*_1_ is assigned a value of 0.5	0.50
*G*_2_	Government credibility loss	Lee et al. (51) highlight that public trust in government information constitutes a critical boundary condition in risk communication, and a lack of trust significantly reduces public compliance with protective governance measures. Li et al. (52) further demonstrate from a trust heterogeneity perspective that under low-trust conditions, public negative responses to environmental risks are markedly amplified, and this effect is persistent, exhibiting asymmetric reputational depreciation. Accordingly, in this study, the government's credibility loss under regulatory failure is set to be ten times the credibility gain (*G*_2_ = 10 × *G*_1_)	5

**Figure 3:**
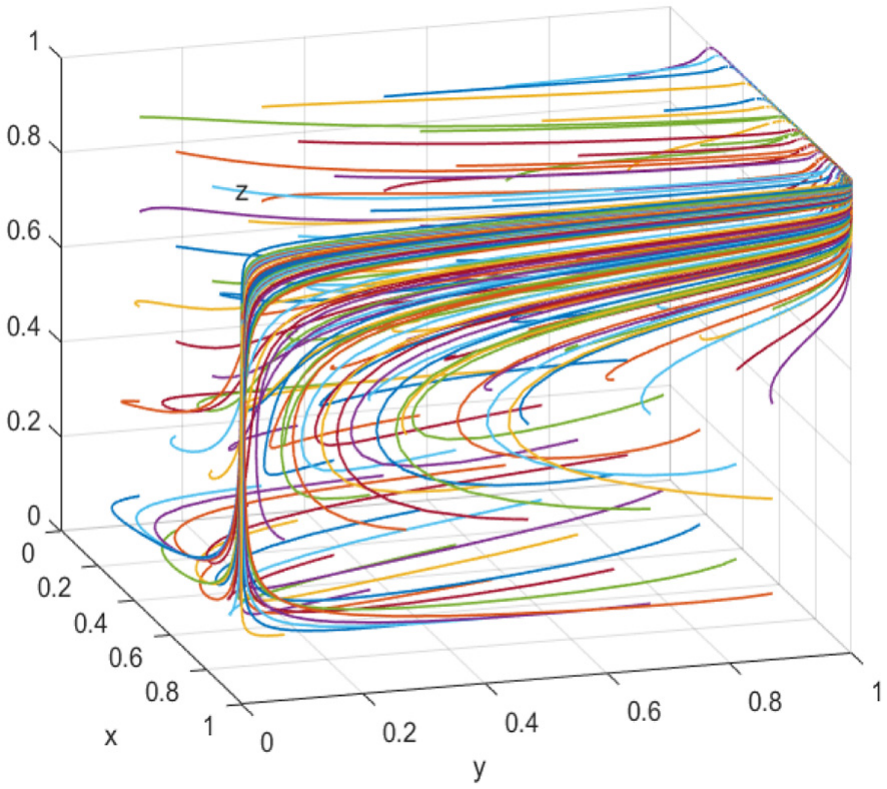
Evolutionary path of tripartite strategy (single column).

To explore the conditions under which the tripartite evolutionary game reaches the ideal equilibrium state of (1, 1, 1), this study first analyzes how variations in key influencing factors at this ideal equilibrium affect the behavioral choices of the three actors—government, specialized straw cooperatives, and farmers. The analytical results under the ideal state are then used as a benchmark for subsequent simulations under realistic scenarios, enabling a clear comparison between ideal and actual settings in terms of actors' strategic choices and their underlying drivers. Numerical programming and simulation analyses are conducted using MATLAB R2016b to systematically investigate the dynamic effects of changes in core influencing factors—such as the intensity of policy incentives, the costs of straw collection, storage, and transportation, and the economic returns from farmers' participation—on the strategic evolution of the three actors. As indicated by the simulation results shown in Figure 3, under China's current policy framework, the evolutionary game system ultimately converges to the (1, 1, 1) evolutionary stable strategy (ESS). Specifically, the government tends to adopt a regulatory strategy, specialized straw cooperatives choose to provide straw CS&T services, and farmers opt to participate by accepting the straw disposal services offered by these cooperatives.

### 5.2 Sensitivity analysis of key variables

It is assumed that the initial participation willingness of the government, straw-specialized cooperatives, and farmers is 0.5, i.e., *x* = *y* = *z* = 0.5. Subsequently, by varying the values of key parameters such as *R*_1_, *S*_1_, *S*_2_, etc., the impacts of changes in these factors on the evolutionary game process and outcomes are examined.

**Figure 4:**
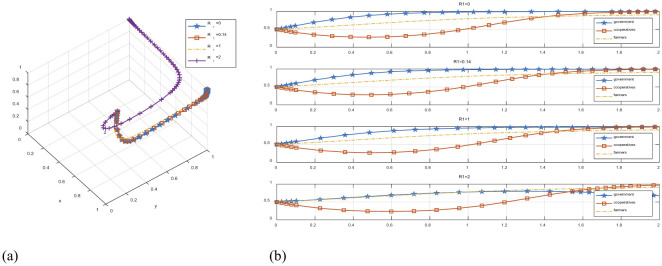
Impact of parameter *R*_1_ (regulatory costs; two columns). **(a)** Phase diagram of evolutionary trajectories under *R*_1_. **(b)** Evolutionary dynamics of the game under different *R*_1_ values.

**Figure 5:**
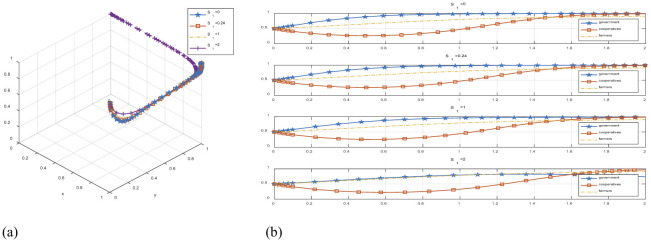
Impact of parameter *S*_1_ (basic subsidies for straw-specialized cooperatives; two columns). **(a)** Phase diagram of evolutionary trajectories under *S*_1_. **(b)** Evolutionary dynamics of the game under different *S*_1_ values.

**Figure 6:**
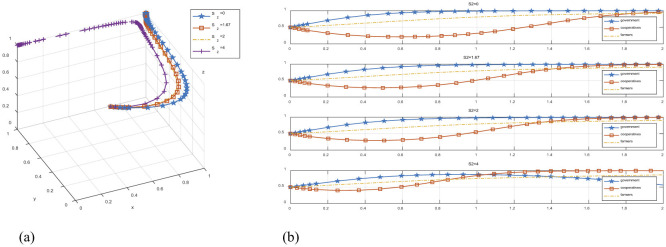
Impact of parameter *S*_2_ (government subsidies to straw-specialized cooperatives; two columns). **(a)** Phase diagram of evolutionary trajectories under *S*_2_. **(b)** Evolutionary dynamics of the game under different *S*_2_ values.

**Figure 7:**
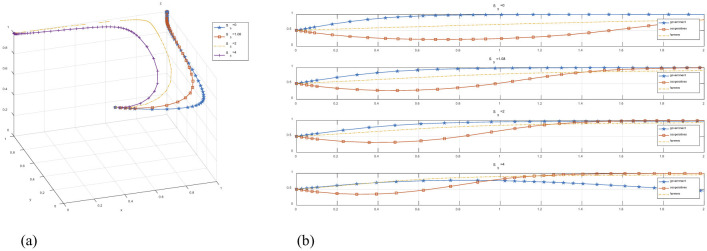
Impact of parameter *S*_5_ (government subsidies to farmers; two columns). **(a)** Phase diagram of evolutionary trajectories under *S*_5._ **(b)** Evolutionary dynamics of the game under different *S*_5_ values.

**Figure 8:**
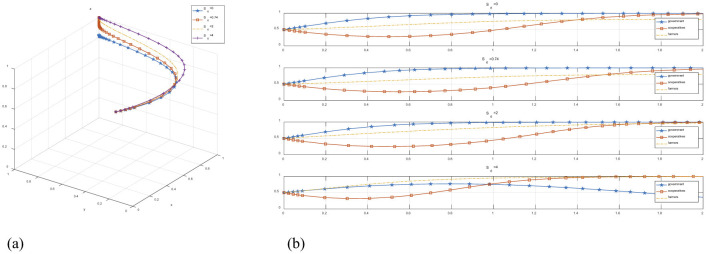
Impact of parameter *S*_6_ (subsidies to farmers from cooperatives; two columns). **(a)** Phase diagram of evolutionary trajectories under *S*_6_. **(b)** Evolutionary dynamics of the game under different *S*_6_ values.

**Figure 9:**
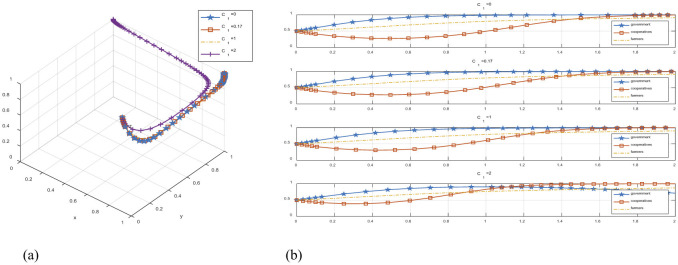
Impact of parameter *C*_1_ (incentives for cooperatives; two columns). **(a)** Phase diagram of evolutionary trajectories under *C*_1_. **(b)** Evolutionary dynamics of the game under different *C*_1_ values.

**Figure 10:**
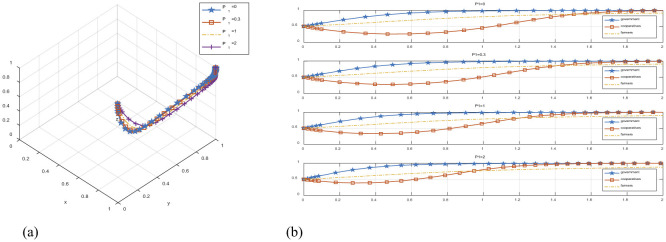
Impact of parameter *P*_1_ (government fines for negatively managed cooperatives; two columns). **(a)** Phase diagram of evolutionary trajectories under *P*_1_. **(b)** Evolutionary dynamics of the game under different *P*_1_ values.

**Figure 11:**
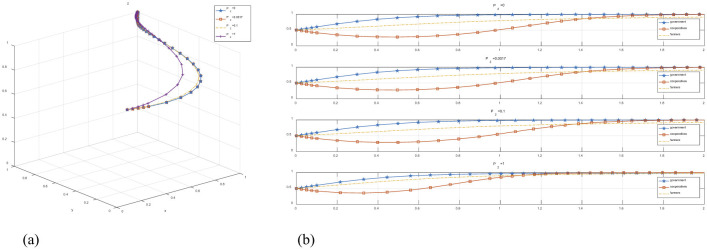
Impact of parameter *P*_2_ (farmers' fines; two columns). **(a)** Phase diagram of evolutionary trajectories under *P*_2_. **(b)** Evolutionary dynamics of the game under different *P*_2_ values.

**Figure 12:**
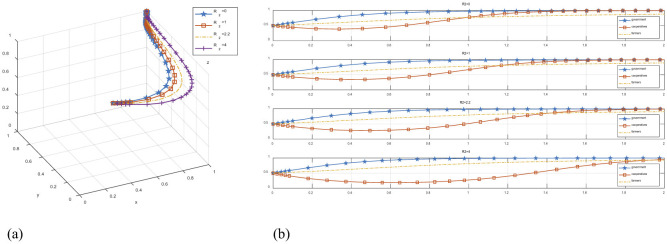
Impact of parameter *R*_2_ (other costs of straw CS&T by straw-specialized cooperatives; two columns). **(a)** Phase diagram of evolutionary trajectories under *R*_3_. **(b)** Evolutionary dynamics of the game under different *R*_3_ values.

**Figure 13:**
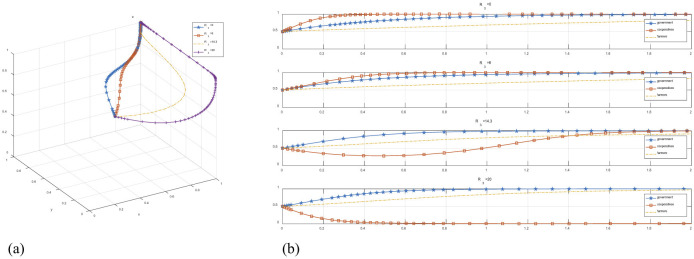
Impact of parameter *R*_3_ (costs of straw CS&T by straw specialized cooperation; two columns). **(a)** Phase diagram of evolutionary trajectories under *R*_3_. **(b)** Evolutionary dynamics of the game under different *R*_3_ values.

**Figure 14:**
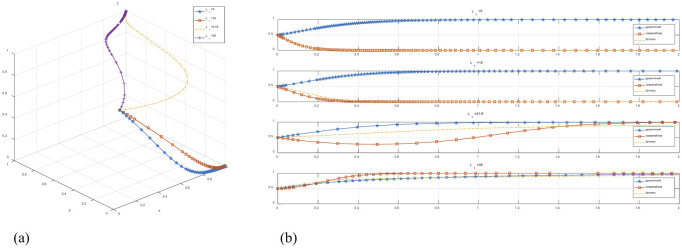
Impact of parameter *L*_1_ (gain to farmers; two columns). **(a)** Phase diagram of evolutionary trajectories under *L*_1_. **(b)** Evolutionary dynamics of the game under different *L*_1_ values.

- In order to analyze the impact of changes in regulatory cost *R*_1_ on the process and outcome of the evolutionary game, *R*_1_ is assigned *R*_1_ = 0, 0.14, 1, and 2, respectively, and the simulation results of replicating the system of dynamic equations evolving 50 times are shown in Figure 4. Figure 4a shows that there exists a threshold $R_{1}^{*}$ (1 < $R_{1}^{*}$ < 2), and the stabilization point is the Pareto equilibrium state (1, 1, 1) when *R*_1_<$R_{1}^{*}$, i.e., the government regulates, the straw-specialized cooperatives provide straw CS&T services, and the farmers sell straw. When *R*_1_ > $R_{1}^{*}$, the system stabilization point deviates to (0, 1, 1). At this point, the government does not regulate; straw-specialized cooperatives provide straw CS&T services; farmers sell straw. This is because the high cost of regulation increases the financial burden of the government. Thus, the government prefers not to regulate. As *R*_1_ increases, the probability of government regulation gradually decreases (see Figure 4b), indicating that excessively high regulatory costs can undermine the government's leading role in rural public health governance. This, in turn, elevates the risk of open straw burning, directly threatening air quality and residents' respiratory health. Therefore, optimizing regulatory mechanisms to reduce administrative costs is a prerequisite for ensuring the long-term effectiveness of public health risk control.
- The impact of changes in the basic subsidy S_1_ provided by the government to straw-specialized cooperatives on the evolutionary game process is analyzed by setting *S*_1_ = 0, 0.24, 1, 2. The simulation results of the replicated dynamic equations over 50 time steps are shown in Figure 5. When *S*_1_ = 0, the model represents an extreme policy scenario in which government subsidies are completely withdrawn. In this case, the probability of cooperatives choosing to implement CS&T services evolves very slowly, indicating a high dependence of the system on policy incentives at the early stage of development. Figure 5a shows that there exists a threshold $S_{1}^{*}$ (1 < $S_{1}^{*}$ < 2). When *S*_1_<$S_{1}^{*}$, the stable equilibrium converges to the Pareto optimal state (1, 1, 1). When *S*_1_<$S_{1}^{*}$, the system shifts to a stable point of (0, 1, 1), where the government chooses not to regulate. This is because higher regulatory costs increase the fiscal burden on the government, making non-regulation a more favorable strategy. As *S*_1_ increases, the probability that straw-specialized cooperatives choose to provide CS&T services in the early stage also increases (Figure 5b). This is because higher government subsidies increase the cooperatives' expected returns, making them more willing to engage in CS&T activities. Therefore, the government needs to set an appropriate subsidy level to encourage cooperatives to establish standardized collection and storage systems, facilitating the transition from decentralized straw accumulation to centralized management. This helps prevent the public health risks associated with mold growth affecting rural water sources and living environments at the source.
- Effects of changes in government subsidies for agricultural machinery purchases *S*_2_ granted to straw-specialized cooperatives on the evolutionary game process. The subsidy parameter was set at *S*_2_ = 0, 1.67, 2, and 4, respectively. Based on these values, the replicator dynamic equations were simulated over 50 evolutionary iterations, with the corresponding results illustrated in Figure 6. When *S*_2_ = 0, the model simulates an extreme policy scenario in which the government provides no subsidies for agricultural machinery purchases. Under this condition, straw-specialized cooperatives lack sufficient incentives to actively provide CS&T services due to the absence of machinery subsidies, resulting in a substantial delay in the establishment and development of the CS&T system compared with expected targets. As shown in Figure 6a, when *S*_2_<$S_{2}^{*}$ (2 < *S*_2_ < 4), the system converges to the Pareto-optimal equilibrium point (1, 1, 1), indicating that the government adopts a regulatory strategy, straw-specialized cooperatives engage in straw CS&T activities, and farmers choose to accept the services provided by the cooperatives. When *S*_2_ > $S_{2}^{*}$, the stable equilibrium shifts to (0, 1, 1), under which the government opts for non-regulation, while straw-specialized cooperatives continue to carry out straw CS&T, and farmers remain willing to participate. As *S*_2_ increases, the probability that straw-specialized cooperatives engage in straw CS&T during the early stages of the evolutionary process rises significantly (Figure 6b). This is because higher government subsidies enhance the expected returns of straw-specialized cooperatives, thereby strengthening their incentives to participate in straw handling activities. Furthermore, subsidies for agricultural machinery accelerate the adoption of specialized off-field operation equipment, enhancing straw transportation efficiency, reducing the residence time of agricultural residues in fields, and strengthening rural public health and epidemic prevention capacity. Therefore, the government needs to select appropriate subsidy levels to incentivize cooperative participation, while ensuring that investment remains proportionate to the expected returns.
- In order to analyze the influence of the change of *S*_5_, the subsidy given by the government to farmers for straw disposal, on the process and results of the evolutionary game, *S*_5_ is assigned as *S*_5_ = 0, 1.08, 2, 4, and the simulation results are shown in Figure 7. When *S*_5_ = 0, the model simulates an extreme scenario in which the government completely withdraws subsidies for farmers. The simulation results show that, in the absence of direct economic incentives, the evolutionary speed at which farmers choose to accept CS&T services decreases significantly. This finding demonstrates that cash subsidies at the initial stage are a critical lever for changing farmers' traditional straw disposal practices. Figure 7a shows that the stabilization point is the Pareto equilibrium (1, 1, 1) when *S*_5_<$S_{5}^{*}$ (1.08 < *S*_5_ < 2). When *S*_5_ > $S_{5}^{*}$ the system stability point deviates to (0, 1, 1) and the government becomes non-regulatory. As S_5_ increases, the probability of farmers choosing to sell straw increases (Figure 7b). This is due to the fact that farmers' income rises as a result of the increase in subsidies, and farmers tend to accept the sale of straw in order to receive subsidies from the government. Therefore, the government should scientifically determine an appropriate subsidy level that can enhance farmers' participation incentives while also ensuring the sustainability of fiscal expenditure. Through targeted economic incentives, farmers can be encouraged to abandon open-field burning practices, which can substantially reduce the threat of air pollution to rural public health. In this way, the coordinated goals of environmental improvement and rural health protection can be achieved.
- In order to analyze the influence of the change of the subsidy S_6_ given to farmers by the straw specialized cooperative on the process and the result of the evolutionary game, *S*_6_ is assigned *S*_6_ = 0, 0.74, 2, 4, and the simulation results are shown in Figure 8. When *S*_6_ = 0, the model simulates a market-deficiency scenario in which cooperatives provide no recycling incentives to farmers. The simulation results indicate that, in the absence of a market-based benefit-sharing mechanism, the system fails to reach an ideal stable equilibrium. Figure 8a shows that there is a threshold $S_{6}^{*}$ (0 < *S*6 < 0.74). The stabilization point is the Pareto equilibrium (1, 1, 1) when *S*_6_ > $S_{6}^{*}$, and the system does not reach the stabilization state when *S*_6_<$S_{6}^{*}$. Take *S*_6_ = 0 as an example, there exists an equilibrium point (1, 1, z^*^), but this equilibrium point is not stable, at this time, the government chooses to regulate, and the straw professional cooperative carries out straw CS&T, but the farmers have about 75% probability of choosing to sell straw. The larger *S*_6_ is, the higher the probability that farmers sell straw, the lower the probability that straw-specialized cooperatives carry out straw CS&T, and the lower the probability that the government chooses to supervise them (Figure 8b). From the perspective of public policy, this finding reflects the complementary relationship between market incentives and administrative regulation. When cooperatives increase subsidies and make straw participation economically beneficial for farmers, market mechanisms can spontaneously encourage farmer participation. Under such conditions, the government no longer needs to maintain intensive supervision and inspection in order to sustain the stable operation of the governance system.
- In order to analyze the influence of the change of the bonus *C*_1_ given by the government to the straw-specialized cooperatives on the process and results of the evolutionary game, *C*_1_ is assigned to *C*_1_ = 0, 0.17, 1, 2, and the simulation results are shown in Figure 9. From Figure 9, it can be seen that the effect of *C*_1_ change on the evolutionary game is almost the same as that of the government subsidies for straw cooperatives such as *S*_1_ mentioned above, which is because both the subsidies and bonuses belong to the incentive funds for straw cooperatives. However, in practice, there is a difference between subsidies and bonuses. Subsidies are given as incentives during the implementation process of the straw-specialized cooperatives, while bonuses are given as incentives after the implementation of the specialized straw cooperatives based on the effect of the implementation of the straw-specialized cooperatives. From a public policy perspective, a performance-based reward mechanism can effectively reduce fiscal risks. By linking financial rewards to actual governance outcomes, the government can motivate cooperatives to ensure thorough straw disposal, prevent opportunistic subsidy-seeking behavior, and eliminate governance blind spots. Therefore, an incentive system that combines upfront subsidies with outcome-based rewards should be established to ensure substantial and lasting improvements in the rural public health environment.
- In order to analyze the effect of the change of government fines on negatively managed straw-specialized cooperatives on the process and outcome of the evolutionary game, *P*_1_ was assigned *P*_1_ = 0, 0.3, 1, 2, and the simulation results are shown in Figure 10. Figure 10a shows that the change of P_1_ does not affect the final evolution results, and the final stabilization points are all (1, 1, 1). However, the change of *P*_1_ affects the evolution speed of the participants' strategy choice. The larger *P*_1_ is, the slower the government chooses to regulate, and the faster the straw specialized cooperative carries out the straw CS&T (Figure 10b). Therefore, if the government intends to reduce penalties imposed on CS&T cooperatives, it must strengthen regulatory oversight. Conversely, the government can appropriately increase penalties to incentivize cooperatives to engage in straw collection, storage, and transportation, ensuring strict compliance with hygiene and environmental protection standards throughout the CS&T process.
- In order to analyze the effect of the change of the government's fines for farmers who conduct straw burning and randomly discard on the process and outcome of the evolutionary game, *P*_2_ was assigned *P*_2_ = 0, 0.0017, 0.1, and 1, and the simulation results are shown in Figure 11. Figure 11a shows that changes in *P*_2_ do not affect the final evolutionary outcome, and the final equilibrium points are all (1, 1, 1). However, it affects the speed of evolution of participants' strategic choices, with farmers selling straw faster and the government choosing to regulate slower as *P*_2_ increases (Figure 11b). Therefore, if the government wants to reduce the fines for farmers, the government must strengthen its supervision. The government can increase the fines for farmers to encourage them to sell straw. However, in practice, the Heilongjiang Provincial Ecological and Environmental Protection Comprehensive Administrative Law Enforcement Bureau said: “According to the relevant provisions of the *Law on Prevention and Control of Air Pollution*, you can burn straw in the open and cause pollution of the penalty of 500 yuan to 2,000 yuan, but our country is mainly to guide the education of the main, the burning of straw farmers themselves and no actual penalties.”
- The impact of changes in the other costs of straw CS&T by straw-specialized cooperatives on the evolutionary game process was analyzed by assigning *R*_2_ values of 0, 1, 2.2, and 4. The simulation results of the dynamic system equations over 50 iterations are shown in Figure 12. Figure 12a indicates that changes in *R*_2_ do not affect the final evolutionary outcome, with all stable points remaining at the Pareto equilibrium (1, 1, 1), but they significantly influence the evolution speed. As *R*_2_ decreases, the rate at which straw-specialized cooperatives carry out CS&T increases, while the speed of government supervision decreases (see Figure 12b). Therefore, if the other costs for straw-specialized cooperatives decrease, they are more inclined to perform straw CS&T, allowing the government to appropriately relax regulatory efforts.
- In order to analyze the influence of changes in other costs of straw CS&T by straw specialized cooperation on the process and results of the evolutionary game, *R*_3_ was assigned *R*_3_ = 0, 6, 14.3, 20, and the simulation results are shown in Figure 13. Figure 13a shows that the variation of *R*_3_ does not affect the final evolution results, and the stabilization points are all (1, 1, 1), but significantly affects the evolution rate. With the decrease of *R*_3_, the straw specialized cooperative society carries out straw CS&T faster, and the government chooses to regulate it slower (Figure 13b). Optimizing operational costs enhances the sustainability of public health governance, allowing the environmental defense system to remain stable even under weak regulatory conditions. Therefore, if the other costs of straw CS&T by straw-specialized cooperatives are reduced, straw-specialized cooperatives will be more inclined to carry out straw CS&T, and the government can appropriately relax the regulation.
- In order to analyze the influence of the change of the gain *L*_1_ from the sale of straw on the process and results of the evolutionary game, *L*_1_ was assigned *L*_1_ = 0, 10, 21.6, 30, and the simulation results are shown in Figure 14. Figure 14a shows that there is a threshold $L_{1}^{*}$ (10 < $L_{1}^{*}$ < 21.6), and the stability point is (1, 1, 1) when *L*_1_ > $L_{1}^{*}$. When *L*_1_<$L_{1}^{*}$, the stability point of the system deviates to (1, 0, 0), at which time the government chooses to regulate, the straw-specialized cooperatives do not carry out the straw collection and storage run, and the farmers do not sell the straw. This is due to the fact that the return from the sale of straw is too small to be attractive to the straw cooperatives and farmers, so the probability of the straw cooperatives CS&T straw and the probability of the farmers selling straw tends to be 0. With the increase of *L*_1_, the rate of straw cooperatives CS&T straw and the rate of farmers selling straw accelerate (Figure 14b). Therefore, if revenue from straw sales increases, both CS&T cooperatives and farmers will be significantly more motivated to participate in off-field straw collection, providing an economic foundation for establishing a hygienic and modernized rural ecological environment.

## 6 Conclusions

This study comprehensively considers the strategic interactions among the government, cooperatives, and farmers with respect to rural public health security, compliant operations, and individual economic benefits. The study contributes in two main aspects. First, at the theoretical level, it integrates straw resource utilization with rural public health prevention and control, establishing a dual-objective governance framework. Second, at the methodological level, it employs field survey data from 30 key counties in China for parameter calibration, which improves the practical relevance and empirical applicability of the simulation results. Based on evolutionary game analysis and numerical simulation, the main conclusions are as follows:

The strategic choices of the three stakeholders exhibit strong interdependence and dynamic linkage. According to the results derived from the replicator dynamic equations, the evolutionary proportion of the government's regulatory strategy does not evolve independently. Instead, it is positively associated with both the proportion of straw-specialized cooperatives providing straw removal services through CS&T and the proportion of farmers willing to accept these services. When cooperatives actively participate in straw CS&T, and farmers respond by cooperating with these services, the marginal benefits of government regulation increase, which raises the likelihood of the government adopting a regulatory strategy. In contrast, when cooperatives engage passively and farmers refuse to cooperate, regulatory cost pressures on the government intensify, increasing the probability of regulatory withdrawal. Similarly, the proportion of cooperatives choosing to implement straw removal services is jointly influenced by the strength of government regulation and farmers' willingness to participate. Strong regulatory enforcement and effective subsidy policies enhance the income stability of compliant operations, which significantly increases cooperatives' incentives to provide CS&T services. Farmers' decisions to accept cooperative services depend on both regulatory policy signals and service quality. Effective government regulation reduces the risk of penalties associated with non-compliant straw disposal, while accessible services and well-designed subsidies further strengthen farmers' willingness to participate. From a public policy perspective, this finding highlights the fiscal boundary for the sustainable operation of governance policies. The ideal governance outcome cannot be achieved solely through higher expenditures or stronger regulation. Fiscal sustainability is also necessary. Only when the social and public benefits generated by governance are sufficient to offset administrative costs can the government maintain policy continuity. This condition provides the incentive for sustained implementation of policies. It also supports the formation of a stable, long-term interaction pattern between cooperatives and farmers.

The model reaches the ideal evolutionary stable equilibrium of government regulation, cooperative implementation of CS&T services, and farmer participation only under certain key conditions. From the evolutionary stability analysis, the core requirement for achieving this equilibrium is that the net benefit of government regulation must be greater than the sum of regulatory costs and policy incentive expenditures. Specifically, the combined credibility gains from government regulation and the credibility losses incurred when the government does not regulate must exceed the total costs of regulation, including subsidies and reward payments to straw-specialized cooperatives, as well as targeted subsidies provided to farmers. From a public policy perspective, this finding highlights the fiscal boundary that ensures the stable operation of governance policies. The ideal state cannot be achieved simply by increasing public expenditure or strengthening regulatory intensity. Fiscal sustainability is essential. Only when the social and public benefits generated by governance can cover administrative costs will the government have sufficient incentive to maintain policy continuity. This condition supports the formation of a long-term and stable interaction pattern between cooperatives and farmers.

Different influencing factors exhibit heterogeneous effects on the evolutionary trajectories of the three actors' strategies and on the location of the system's stable equilibrium, with positive incentives proving more effective than punitive measures. The numerical simulation results reveal three key findings. First, under current conditions, government regulatory strategies exert a dominant influence on the behavioral decisions of both straw-specialized cooperatives and farmers. Given the limited scale of existing straw CS&T systems and the persistence of traditional disposal practices, the presence and intensity of government regulation directly shape the compliance risk costs faced by cooperatives and the violation costs associated with farmers' extensive disposal behaviors. These factors significantly affect the willingness of both parties to participate in CS&T activities. As the straw industry chain becomes more mature in the future, supported by increasing market revenues and reductions in both cooperatives' operating costs and farmers' disposal costs, the endogenous motivation of these actors to participate in straw handling will strengthen. In such circumstances, the dominant role of government regulation and incentives is expected to gradually decline, while market mechanisms and actors' self-discipline will assume a more prominent role in sustaining system stability. Second, incentive-based factors exert a stronger influence on the system's stable equilibrium and the strategic choices of the three actors. Adjustments in government subsidies and performance-based rewards for straw-specialized cooperatives, targeted subsidies for farmers, and recycling payments from cooperatives to farmers directly reshape the payoff structures of all three parties. These changes can shift the system's evolutionary stable equilibrium and guide strategic behavior. Third, penalty-based factors have a relatively limited effect on system stability and decision-making. Changes in fines imposed on cooperatives for non-compliant operations and on farmers for extensive straw disposal increase violation costs to some extent, but do not fundamentally alter the underlying payoff balance among the three actors. As a result, they do not change the core evolutionary stable equilibrium of the system, and their influence on strategic choices remains weaker than that of positive incentives. This finding has important policy implications. When designing policy schemes, the government should prioritize the establishment of a precise positive incentive system. A combination of more rewards and fewer penalties can effectively stimulate the enthusiasm of cooperatives and farmers. This approach enables the achievement of both straw resource utilization and rural public health governance objectives at relatively low cost.

## 7 Discussion and policy implications

Building on the tripartite evolutionary game analysis, evolutionary stability assessment, and numerical simulation results presented above, and in consideration of current policy orientations and practical conditions of straw CS&T, this section offers an in-depth discussion and proposes targeted optimization strategies. By focusing on the functional roles and interest demands of the three core stakeholders—the government, farmers, and straw-specialized cooperatives—and addressing key objectives such as improving the efficiency of off-field straw utilization, strengthening rural public health risk prevention, and promoting coordinated governance among the three parties, this section seeks to provide policy-relevant insights for enhancing the effectiveness and quality of straw off-field utilization.

The conclusions of this study are broadly consistent with existing research on agricultural ecological governance, which suggests that positive incentives are more effective than purely punitive mechanisms in guiding participation behavior among farmers and cooperatives (45). However, unlike studies that regard government regulatory intensity as a single dominant factor, this study finds a clear dynamic interaction among government regulation, standardized cooperative operation, and active farmer participation. Strategy adjustments by a single actor are insufficient to drive stable system evolution on their own. Only a multi-stakeholder coordinated governance mechanism can ensure the long-term stability of standardized straw off-field utilization and public health risk control (39). In addition, the sensitivity analysis further indicates significant differences in how various policy instruments affect the system's evolutionary path and convergence speed. Subsidy policies mainly influence participation probabilities and equilibrium stability, while punitive mechanisms have a stronger effect on the speed at which the system converges to stability. This suggests that straw governance policies should not only focus on policy intensity, but also consider the structure of policy mixes and differences across implementation stages.

At the government level, a policy system that combines short-term incentives with long-term institutional arrangements should be established. In the straw CS&T system, the government plays core roles in institutional supply, fiscal support, and regulatory guidance. Based on the model results and sensitivity analysis, short-term policies should focus on increasing the participation incentives of cooperatives and farmers through targeted subsidies, reducing the threshold for the system to reach a stable equilibrium. In the long run, the CS&T system should gradually move toward institutionalization, market-oriented operation, and regionally coordinated governance. First, the fiscal subsidy structure should be optimized to improve the precision of fund allocation. Sensitivity analysis shows that when government subsidies to cooperatives remain within the range of 1–2, cooperatives exhibit the highest probability of evolving toward active participation. When agricultural machinery subsidies remain within the range of 2–4, they effectively reduce cooperative operating costs and enhance the willingness for large-scale operation. Therefore, subsidy standards should be dynamically adjusted according to regional straw resource endowments, transportation distances, and local fiscal capacity, avoiding uniform subsidy policies. In addition, subsidy mechanisms should gradually shift from universal subsidies to performance-oriented subsidies. Indicators such as standardized straw off-field utilization rates, open-field burning control rates, and public health risk prevention outcomes should be incorporated into fiscal performance evaluation systems to improve the efficiency of fund utilization. Second, regulatory and enforcement costs should be properly controlled. The model results indicate that although higher penalties do not alter the final equilibrium outcome, they can significantly accelerate the speed of strategic evolution. Therefore, under conditions of limited administrative resources, local governments should not rely excessively on high-intensity punishment. Instead, penalty standards should be set according to actual regulatory capacity, with emphasis placed on a governance approach that combines moderate punishment with positive incentives. This helps create stable policy expectations and reduce long-term governance costs. At the same time, grassroots regulatory capacity should be strengthened. Township governments and village-level organizations should improve their implementation capacity in straw-burning inspections, policy communication, and information feedback to avoid policy deviations caused by weak local administrative capacity. Third, greater attention should be given to technology promotion and public service provision. The parameter analysis indicates that technologies such as straw baling, crushing, storage, and intelligent dispatching can effectively reduce cooperative operating costs and improve CS&T efficiency (46). In the short term, the government should strengthen support for agricultural machinery and technical training. In the long term, efforts should focus on developing digital straw management platforms and gradually establishing cross-regional straw allocation and information-sharing mechanisms to improve resource allocation efficiency. Fourth, institutional coordination and fiscal sustainability should be strengthened. The straw CS&T system has clear public governance attributes. Its long-term stable operation depends not only on fiscal subsidies, but also on institutional coordination and policy integration. Therefore, the government should coordinate departments related to agriculture and rural affairs, ecological environment, and public finance to establish cross-department governance mechanisms. At the same time, diversified investment models that combine fiscal subsidies, market returns, and social capital participation should be explored to reduce the long-term fiscal burden of high-intensity subsidies and improve the sustainability of the governance system.

At the cooperative level, greater emphasis should be placed on strengthening intermediary functions and long-term coordination capacity. Straw-specialized cooperatives serve as key intermediaries linking governments and farmers, and their operational capacity directly affects the efficiency and stability of the straw CS&T system. The results indicate that reductions in cooperative operating costs significantly increase the probability of active participation and accelerate the system's convergence toward stable equilibrium. Therefore, cooperatives should focus on improving their levels of organization, specialization, and coordination. First, the layout of straw storage sites and collection networks should be optimized. The study finds that unreasonable storage site distribution and excessively large collection radii substantially increase transportation costs and operational risks (21). Cooperatives should therefore design storage station layouts according to regional planting density and transportation conditions in order to reduce transport distances and logistics costs while improving collection efficiency. Second, long-term and stable cooperation mechanisms should be established. Cooperatives should sign long-term straw recycling agreements with farmers that clearly define key terms such as collection quantity, quality standards, pricing, and collection schedules. This can reduce market uncertainty and operational risk. At the same time, long-term supply relationships should be established with downstream straw utilization enterprises to stabilize sales channels and improve revenue expectations. In addition, village-level organizations can help strengthen trust between cooperatives and farmers, reducing coordination and communication costs. Third, cooperatives should improve their risk response capacity and regional coordination ability. In major grain-producing areas such as the Northeast Black Soil Region, where straw output is large and highly seasonal, cooperatives need stronger centralized collection and storage capacity. In contrast, hilly areas or regions dominated by small-scale farming should adopt smaller and more decentralized collection models. Therefore, different regions should develop differentiated cooperative operating models based on local resource endowments and agricultural production structures in order to improve policy adaptability.

At the farmer level, greater attention should be given to strengthening participation capacity and fostering long-term behavioral identification. Farmers are the direct executors of off-field straw utilization practices, and their strategic choices directly affect both the evolutionary direction of the system and the effectiveness of public health governance. The model results show that increasing farmer subsidies can significantly raise participation probabilities, while moderate penalties can accelerate the speed of strategic evolution. Therefore, policy guidance for farmers should place greater emphasis on long-term cognitive development and interest coordination. First, technical training and risk awareness related to straw utilization should be strengthened. Some farmers have limited understanding of the costs, technical risks, and expected benefits associated with off-field straw utilization, which may lead to resistance toward participation. Therefore, agricultural extension agencies, cooperatives, and village-level organizations should provide differentiated technical training and public awareness programs to improve farmers' understanding of both the economic value and public health significance of straw resource utilization (47). Second, information-sharing and benefit-feedback mechanisms should be improved. Information asymmetry is an important factor restricting farmer participation. Governments and cooperatives should rely on digital platforms, village organizations, and agricultural socialized service systems to provide farmers with timely information on subsidy policies, market prices, technical guidance, and straw collection demand. Such measures can reduce decision-making uncertainty and improve farmer satisfaction with public policies. Third, greater efforts should be made to strengthen farmers' environmental responsibility awareness and cooperative identity. Off-field straw utilization is not only a resource utilization issue, but also closely related to rural environmental governance and public health security. Therefore, village regulations, grassroots publicity campaigns, and demonstration households should be used to gradually strengthen farmers' awareness of ecological responsibility and public health risk prevention. These efforts can help promote the formation of long-term and stable green production behaviors.

A long-term multi-stakeholder collaborative governance mechanism should be established. In summary, achieving a balanced state of joint participation in straw CS&T among the government, straw-specialized cooperatives, and farmers requires the establishment of a multi-stakeholder co-governance mechanism that aligns with practical straw handling operations. This co-governance mechanism for off-field straw utilization comprises five sub-mechanisms: policy assurance, market regulation, information sharing, and technological innovation. Among these, the policy assurance mechanism forms the foundational basis for orderly off-field straw utilization. On one hand, it provides clear regulations and institutional support for the entire process of straw management, ensuring the effective operation of other sub-mechanisms. On the other hand, by implementing effective supervision and scientific management throughout the off-field straw utilization process, it fully mobilizes the enthusiasm of all participating stakeholders and promotes the sustainable and healthy development of the straw utilization sector. The market regulation mechanism creates a favorable market environment for the efficient circulation and value realization of straw resources. Through the interplay of supply-demand dynamics, price incentives, and market competition, straw resources are optimally allocated, facilitating their rational flow and maximizing economic value. The technological innovation mechanism serves as the core driver for improving the quality and efficiency of off-field straw utilization. By enhancing the productivity of straw CS&T, and comprehensive utilization through innovation, it supports the transformation and upgrading of the straw utilization industry. The information sharing mechanism effectively addresses information silos among stakeholders, enabling the government, cooperatives, and farmers to share information resources. This ensures transparency and efficiency in information transmission, reduces redundant efforts, enhances overall operational efficiency, and further stimulates the participation of all stakeholders, particularly farmers. These four sub-mechanisms are closely interrelated, mutually supportive, and operate synergistically. Only through an integrated and coordinated approach can the co-governance mechanism for off-field straw utilization function smoothly and efficiently, ultimately achieving the goal of balanced, collaborative participation in straw collection, storage, and transportation by all three stakeholders.

Limitations and future research directions. This study constructs a three-party evolutionary game model to reveal the strategic interaction logic among the government, CS&T cooperatives, and farmers in the straw collection, storage, and transportation process. However, several limitations remain in terms of research depth and breadth. First, the study primarily focuses on the rational interactions of the three core stakeholders, without incorporating straw utilization enterprises and other potential actors into the endogenous analytical framework, and without fully considering the behavioral heterogeneity of farmers of different scales or cooperatives with varying operational characteristics. Future research could integrate key stakeholders such as straw utilization enterprises, logistics providers, and financial institutions, constructing a multi-agent dynamic evolutionary game model to reveal a more comprehensive strategic interaction logic. Second, constrained by the representativeness of the survey sample, model parameters are largely empirically calibrated based on specific regions (e.g., the Northeast Black Soil Region), and thus do not fully capture spatial heterogeneity across different terrains (hills, plains) or cropping systems. Future studies could combine GIS-based spatial analysis with temporal dynamic simulations to explore optimal off-field resource allocation solutions across regions and time periods. Third, the model in this study mainly analyzes the strategic evolution process under the assumption of bounded rationality. However, the representation of behavioral factors such as farmer risk aversion, social norms, neighborhood effects, and cognitive biases remains relatively limited. In reality, farmers' decisions on whether to participate in off-field straw utilization are influenced not only by economic returns, but also by village social networks, trust in government policies, and the demonstration effects of neighboring farmers' behaviors. Due to the difficulty of obtaining relevant behavioral data and the lack of mature quantitative measurement systems, these behavioral factors were not incorporated into the core parameter system of the present model. Future studies could integrate approaches from behavioral economics, experimental economics, and social network analysis. By introducing factors such as risk preferences, social norm constraints, and behavioral learning mechanisms, future research may further improve the explanatory power of evolutionary game models in real-world decision-making contexts. Fourth, the current study pays limited attention to the long-term dynamic evolution of multi-party games, and does not systematically analyze the complex feedback and non-linear effects of strategies over time. Future research could incorporate stochastic evolutionary game theory to simulate long-term dynamic evolution processes, systematically assessing the sustained impacts of policy incentives, market fluctuations, and technological development on CS&T efficiency and rural public health governance.

## Data Availability

The original contributions presented in the study are included in the article/supplementary material, further inquiries can be directed to the corresponding author.
